# Advancements in silver-based nanocatalysts for organic transformations and other applications: a comprehensive review (2019–2024)

**DOI:** 10.1039/d5ra00336a

**Published:** 2025-05-27

**Authors:** Bhoomi Sachdeva, Nisha, Baby, Khushboo Aggarwal, Aarushi Singh, Kamlesh Kumari, Ramesh Chandra, Snigdha Singh

**Affiliations:** a Drug Discovery & Development Laboratory, Department of Chemistry, University of Delhi Delhi-110007 India ssingh3@chemistry.du.ac.in; b Dr B. R. Ambedkar Centre for Biomedical Research (ACBR), University of Delhi Delhi-110007 India; c Institute of Nanomedical Science (INMS), University of Delhi Delhi-110007 India; d Maharaja Surajmal Brij University Bharatpur 321201 Rajasthan India; e Department of Zoology, University of Delhi Delhi-110007 India; f Department of Chemistry, Ramjas College, University of Delhi Delhi-110007 India

## Abstract

Over time, nanocomposites have revolutionized materials science, offering numerous applications in fields such as catalysis, environmental purification and treatment, biomedicine and various industries. Among these, silver-based nanocomposites are particularly notable for their remarkable stability, reusability, biocompatibility, and multifunctional medicinal properties. Hence, we present a comprehensive summary of recent developments (2019–2024) in silver-based nanomaterials, focusing on their applications across multiple domains, including catalytic organic transformations, biomedical uses, environmental remediation, and industrial sectors such as food packaging, agriculture and textiles. By highlighting recent advancements and emerging trends, we aim to provide a thorough understanding of the role of silver-based nanocomposites in contemporary science and technology, emphasizing their potential to drive innovation across diverse disciplines.

## Abstract 2

Over time, nanocomposites have revolutionized materials science, offering numerous applications in fields such as catalysis, environmental purification and treatment, biomedicine and various industries.

## Graphical abstract

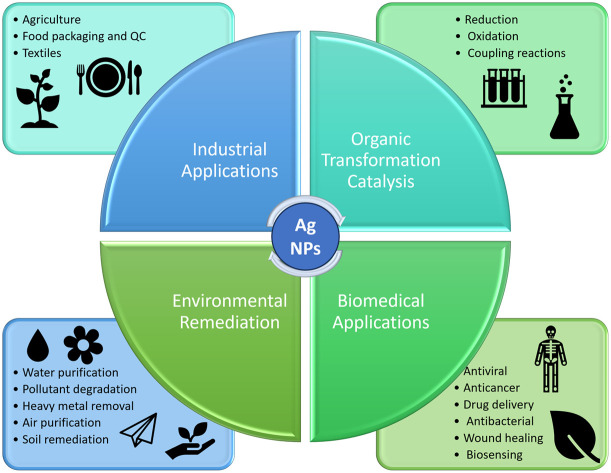

## 1. Introduction

**Fig. 1:**
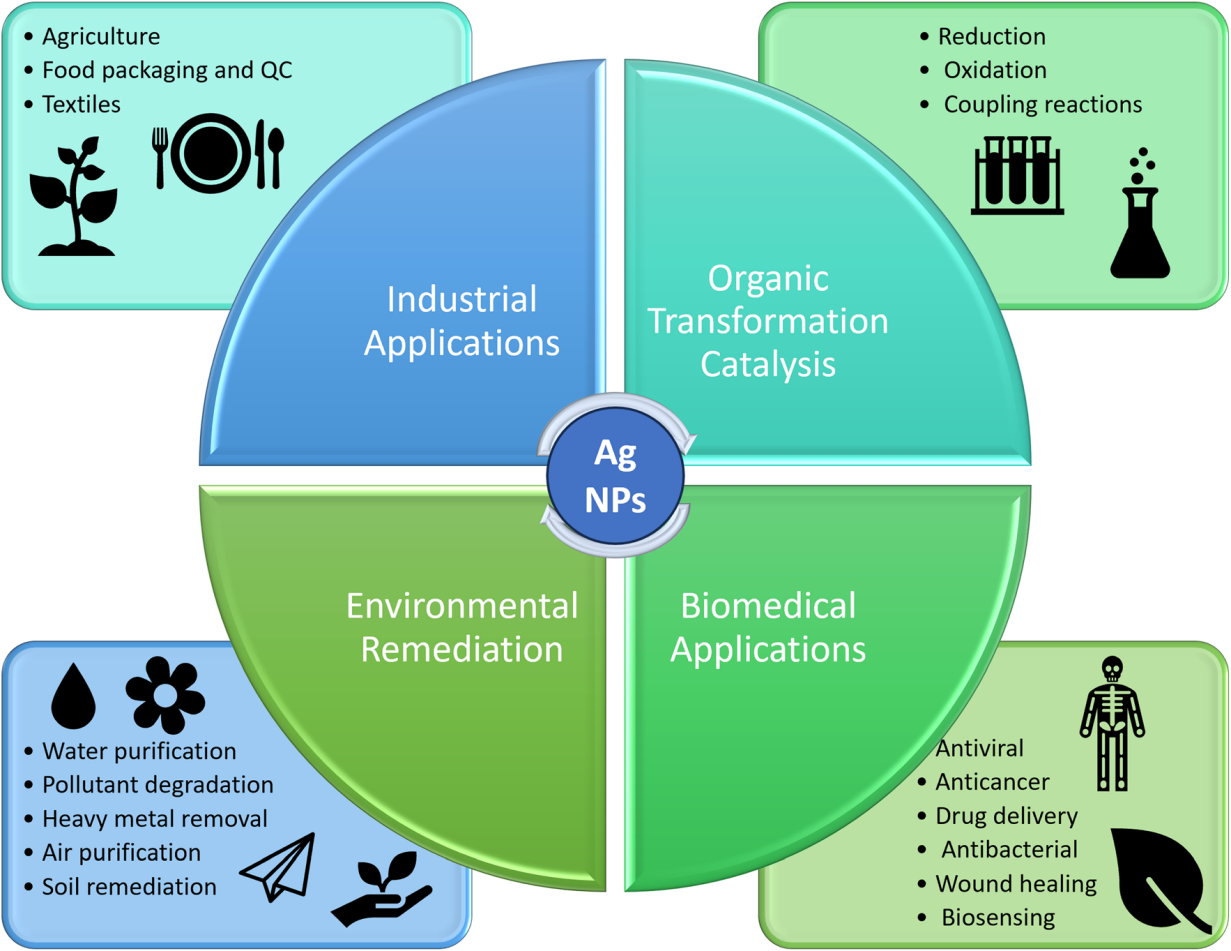
Silver-based nanocomposites and their applications in different fields.

Over recent years, nanocomposites have emerged as transformative materials, offering exceptional mechanical, thermal, and electrical properties due to their high surface area-to-volume ratio which results in higher selectivity and activity compared to conventional materials.^1^ Typically, nanocomposites are formed by dispersing nanoparticles with diverse functionalities within a matrix, making them highly valuable for modern applications in catalysis,^2^ sensing,^3,4^ environmental remediation^5^ and biomedical fields,^6,7^ as well as many more^8,9^ (Fig. 1). In the realm of catalysis, nanocomposites exhibit enhanced catalytic activity emerging from the synergistic interactions between their components, leading to the abundant generation of highly effective active sites.^10,11^ Although homogeneous catalysis is highly efficient, it is hindered by challenges like complex separation, limited recyclability, and potential contamination of the final products. In contrast, heterogeneous catalysis using nanocomposites overcomes these challenges by enabling easy separation, enhanced reusability, and minimal contamination.^12,13^

In catalysis, besides the physical state, several other factors influence the reactivity and selectivity of a catalyst. In general, the number and distribution of active sites on the catalyst influence its catalytic activity, making highly porous materials with a large surface area preferable. In particular, the quantum size effect, shape, morphology and exposed crystalline facets of the catalyst structure significantly influence the binding strength of reactants and intermediates, thereby affecting their activity and selectivity in the reaction.^14,15^ Moreover, the electronic structures of the nanoparticles vary significantly due to the quantum size effect, which also explains their influence on catalytic activity.^14^ Additionally, other nanoscale phenomena such as interatomic distances, coordination numbers, and atomic scale alloying should also be considered.^16^ Furthermore, structural and electronic modifications in a catalyst can arise from heterojunction formation, deposition or the use of a support. For example, metal-supported interactions, whether synergetic or cooperative, can significantly influence the activity and selectivity of the active site.^14^ Researchers can investigate the impact of structural modifications on catalyst reactivity by employing various *in situ* characterization techniques like EPR, XRD and XPS to better understand the changes.^17,18^

Silver nanoparticles (Ag NPs) feature a high surface area relative to their volume and tuneable surface properties. They are widely recognized for their inherent stability, recyclability, and compatibility in several reactions, enhancing their value across multiple applications over time.^19,20^ Over the years, a diverse range of synthetic approaches has been explored, from bottom-up chemical synthesis and top-down physical methods to advanced biological and environmentally friendly techniques.^21,22^ Researchers have successfully controlled the physical properties of nanoparticles, which, in turn, dominate their chemical characteristics. This precise control allowed them to tailor the properties and activities of the resulting nanocomposite, optimizing them for catalyzing specific reactions. Typically, Ag NPs are synthesized using chemical reduction methods with reducing agents like amines, citrates, borohydride, and ascorbic acid. To prevent colloidal agglomeration, the reduction process is carried out in the presence of stabilizing, protecting, or capping agents. Additionally, methods such as hydrothermal, microwave, and photochemical processes have been used to enhance the reduction of ionic Ag^+^ to metallic Ag.^23^

Previous literature surveys have reported that Ag NPs exhibit excellent antibacterial,^24^ antifungal,^24^ antiviral,^25^ antifouling^26^ and antioxidant^27^ properties, which contribute to the biocompatible nature of silver-based nanomaterials. Interestingly, silver being non-toxic, cost effective and environment friendly, has attracted significant attention as a versatile candidate to replace the more expensive or less sustainable metals such as gold, copper, or palladium as precursor materials for various chemical reactions and applications.^28,29^

Among the various nanocatalysts investigated to date, silver-based nanocatalysts have emerged as exceptional,^30^ exhibiting high selectivity and efficiency in facilitating various organic transformations. They include key reactions such as reduction,^31,32^ oxidation,^33^ coupling,^34^ and click reactions^28^ and many more. By now, many researchers have explored different types of silver-based nanocatalysts such as Ag/CeSnO_*x*_,^35^ rGO–Ag NPs,^36^ Ala–Ag NPs,^37^ MWCNTs-NH_2_/Fe_3_O_4_/Ag,^38^ KIT-5-bigua-Ag,^39^ AgNPs@m-PS-PC,^40^ [Ag/Mg_4_Al-LDH],^41^ Fe_3_O_4_@Creatinine@Ag,^42^ ZnO/PANI/Ag,^43^ Ag/MOF,^44^ Fe_3_O_4_@CS-StOX@Cys@Ag^+^,^45^ Ag@mHAp-Si-(S),^46^ Ag@HCMP-bpybph,^47^ and others for promoting organic reactions, offering more sustainable environmental friendly alternatives to conventional chemical processes.

Silver-based nanocomposites are not only extensively used in oxidation, reduction, and coupling processes but also exhibit remarkable versatility in various other chemical transformations. For example, these silver-based nanocatalysts have been crucial in the synthesis of xanthene derivatives, which serve as valuable biological scaffolds as well as spiro compounds, known for their medicinal applications. Additionally, they facilitate biodiesel production *via* the transesterification process and are instrumental in catalyzing the synthesis of quinoline and polyhydroquinoline heterocyclic derivatives, which hold significant medicinal importance.

**Fig. 2:**
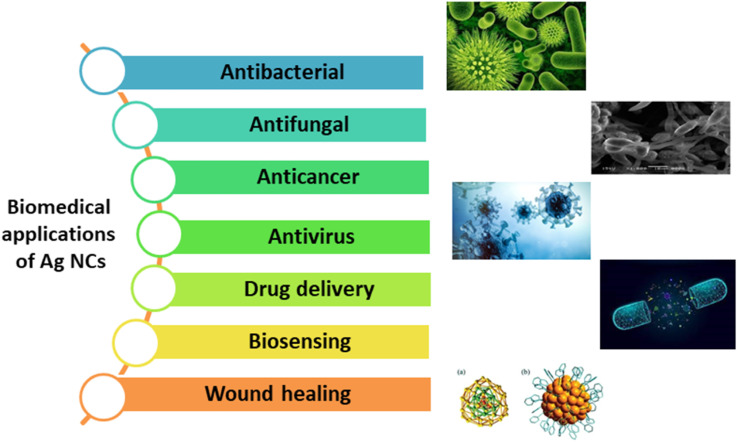
Applications of Silver based nanocomposites particularly in biomedical field.

Moreover, various silver-based nanocatalysts such as [Ag–P(NAA)],^48^ Ag–ZnO/SEP5%,^49^ BML@Ag,^50^ MMT/Fe_3_O_4_/Ag,^51^ Ppy/Ag/Gr,^52^ Ag_2_WO_4_/rGO NCs,^53^ AGCT,^54^ SNC–AgNP^55^ have also effectively addressed various environmental concerns including the degradation of pollutants,^56,57^ wastewater treatment^58–60^ and the conversion of waste materials into valuable products.^61–64^ Ag NPs possess biocompatible antimicrobial, antifouling, and antioxidant properties (Fig. 2), making them an ideal choice for various biomedical applications such as, drug delivery,^65,66^ wound healing,^67,68^ antimicrobial applications and so on.^69–71^ Over the years, researchers have developed various silver-based nanomaterials that efficiently cater to these applications. Additionally, numerous studies highlighted the use of silver-based nanocatalysts in industries, including agriculture,^72^ textiles,^73,74^ and food processing.^75^

In the subsequent sections of this review, we will explore the applications of silver-based nanocatalysts in organic transformations, including, reduction, oxidation, coupling, click reaction and other miscellaneous reactions. We have also highlighted their applications in biomedical, environmental, and industrial fields. By summarizing the latest research findings, we aim to provide a comprehensive overview of the advancements and future prospects of silver-based nanocatalysts in modern science and technology.

## 2. Organic transformations

**Fig. 3:**
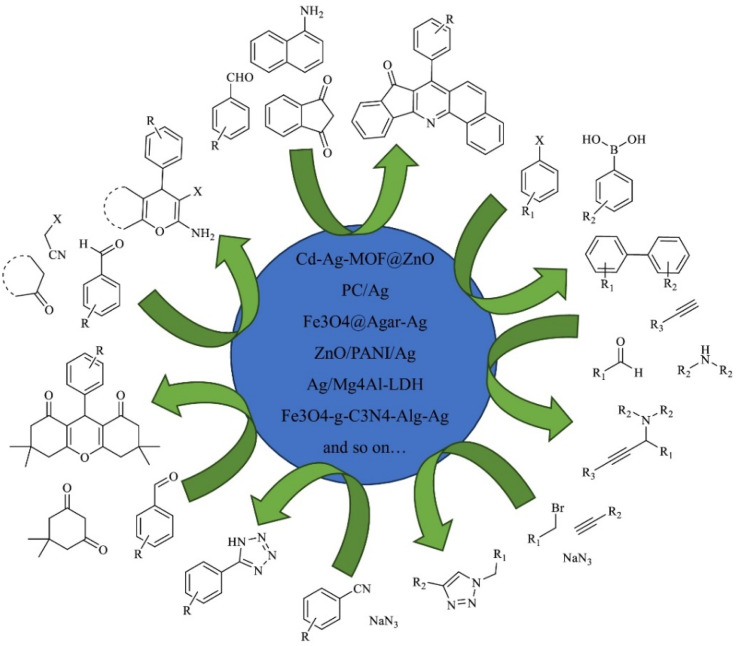
Different organic transformation reactions using silver nanocatalysts.

In this section, we will discuss nano-silver catalyzed organic transformation processes such as reduction, oxidation, coupling reactions, click reactions and more (Fig. 3).

### 2.1 Reduction reactions

#### 2.1.1 Nitroarene reduction

**Scheme 1:**
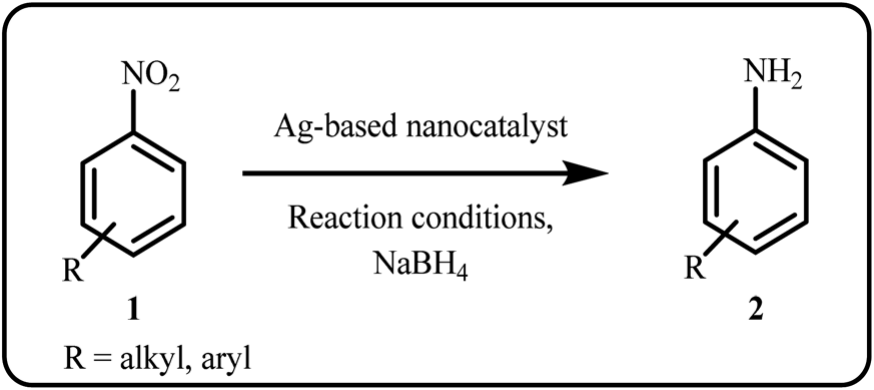
General scheme for reduction of nitroarenes.

The release of nitro group-containing organic compounds into water bodies has anthropogenically polluted our environment, water sources and marine biological systems. Additionally, 4-nitrophenol (4-NP) is widely employed in the synthesis of various insecticides and pesticides, and its continuous use poses significant risks to both human health and the environment. Therefore, reducing hazardous nitro compounds to their amino form presents an environmentally safer alternative. Recently, several research groups have reported the reduction of nitroarenes using silver-based nanoparticles as catalysts in the presence of NaBH_4_ (Scheme 1).^76,77^ Exceptionally, some novel nanocatalysts have demonstrated enhanced reusability, sustaining their efficiency for over 10 to 15 consecutive experimental runs.^46,78^

Recently, several studies have highlighted the use of magnetic nanocatalysts for the reduction of nitroarenes.^79,80^ Khaleghi *et al.* synthesized a novel silver based nanocomposite, Fe_3_O_4_@Cur/Mel-Ag, and evaluated its catalytic activity in the reduction of nitrobenzene derivatives achieving an impressive 98% yield of the corresponding products.^81^ Taheri *et al.*, explained the synergistic interactions between the support and Ag NPs in the designed S-g-C_3_N_4_/starch-Fe_3_O_4_-Ag nanocomposite that exhibited outstanding catalytic activity for nitroarene hydrogenation in presence of NaBH_4_, attaining up to 99% reduction.^82^ Thiol functionalized Fe_3_O_4_ nanoparticles were utilized to immobilize silver nanoparticles, resulting in the fabrication of a magnetically recyclable Fe_3_O_4_/SiO_2_-Pr-S-Ag nanocomposite, which was employed for the catalytic reduction of 4-NP and the degradation of azo dyes.^83^ Subsequently, Veisi *et al.* introduced a Tannic acid coated Fe_3_O_4_ nanoparticles to improve silver ion adsorption and reduction through multiple polyphenol interaction with metal ions, to generate a novel magnetic nanocatalyst, Fe_3_O_4_@TA/Ag which facilitated reduction reactions at room temperature.^84^

Moreover, several studies reported the use of Ag NPs synthesized using green methods for the hydrogenation of different Nitroarenes,^31,85,86^ while numerous others highlights the degradation of organic pollutants using supported Ag NPs.^87^ Mallakpour *et al.* detailed the eco-friendly synthesis of a silver-based nanocatalyst, the CS/Ag-LDH film, for the reduction of 4-NP, achieving approximately 99% conversion in a brief period.^88^ Utilizing lignin as a catalytic support, Xiao *et al.* developed two silver-based nanocatalysts, one with Ag NPs supported on the surface of pre-hydrolyzed lignin (Ag/PL) and other with Ag NPs embedded within lignin (Ag@PL), for the efficient reduction of 4-NP. Ag/PL exhibited higher catalytic efficiency than Ag@PL owing to the increased accessibility and exposure of Ag NPs to reactants.^89^ Han *et al.* developed a silver nanoparticle decorated N-doped reduced graphene oxide nanocatalyst (Ag/N-rGO) for the catalytic reduction of 4-NP.^90^ The catalytic potential of a Cu–Ag bimetallic nanocatalyst (Cu–Ag/PVA), was realized by Wang *et al.* to effectively reduce nitroarene and compared its catalytic efficiency with monometallic Cu or Ag nanocatalyst.^91^ Nguyen *et al.* conducted a fascinating investigation on the use of monomeric and polymeric N-heterocyclic carbenes (NHCs)-functionalized Ag NPs in catalysis reaction.^92^

**Scheme 2:**
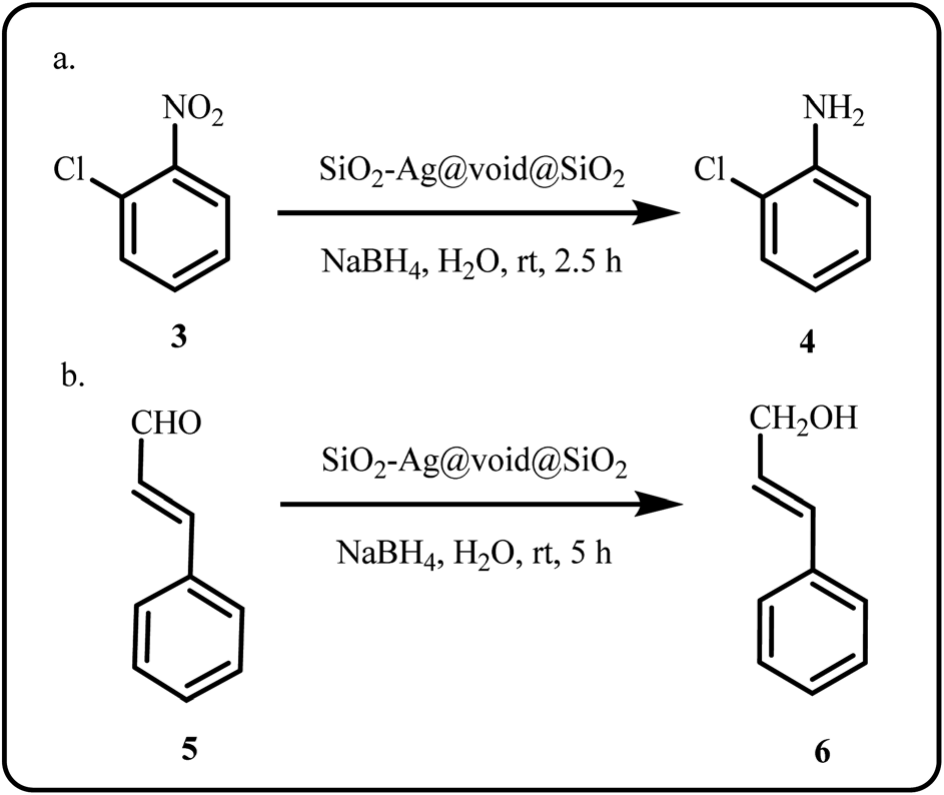
Chemoselective reduction of (a) 2-nitrochlorobenzene and (b) cinnamaldehyde using SiO_2_–Ag@void@SiO_2_ nanocatalyst (Yin *et al.*^93^).

Recently, Yin *et al.* proposed a novel and effective silver-based nanocatalytic system, SiO_2_–Ag@void@SiO_2_, for successful chemoselective hydrogenation of 2-nitrochlorobenzene (2-NCB) and cinnamaldehyde (CA) (Scheme 2). The group encapsulated and uniformly dispersed the prepared Ag NPs within hollow silica, leveraging the micro-reactor and confinement effect of the catalyst to achieve high chemoselectivity.^93^

**Scheme 3:**
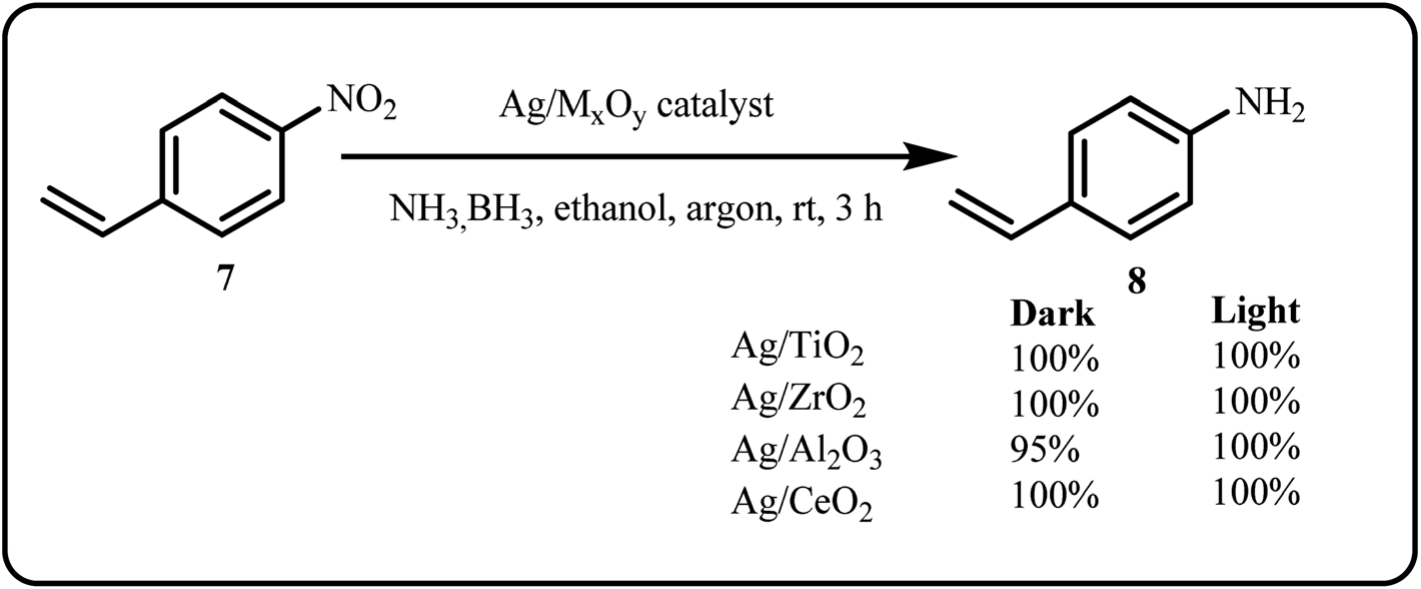
Chemoselective reduction of *p*-nitrostyrene using Ag/M_*x*_O_*y*_ nanocatalyst (Verma *et al.*^95^).

The ternary hybrid, composed of strontium niobate (SrNbO), Ag NPs and nickel–aluminum layered double hydroxide (LDH), was developed by first assembling oppositely charged SrNbO and LDH nanosheets, followed by the *in situ* photo-reduction of silver ions, giving Ag@SrNbO/LDH, which was employed to degrade 4-NP.^94^ Verma *et al.* focused on improving the chemoselective reduction of *p*-nitrostyrene to *p*-aminostyrene (Scheme 3). Amongst all, the Ag/TiO_2_ nanocatalyst outperformed obtaining a 100% conversion and maximum chemoselectivity of 81%. Additionally, the group tested the reaction under different wavelengths of visible light, with green Light Emitting Diode (LED) producing the highest activity.^95^ Several other nanocatalysts used for reduction reactions are summarized in Table 1.

**Table 1:** Comparative analysis of silver-based nanocatalyst for reduction reactions^a^

S. no.	Catalyst	Time (min)	Isolated yield	Reference
1	MWCNTs-NH_2_/Fe_3_O_4_/Ag NPs	30	99	Hamelian *et al.*^38^
2	Fe_3_O_4_@LP-Ag	8	86–100	Ahmad *et al.*^80^
3	rGO–Ag NPs	3	100	Sun *et al.*^36^
4	Cu–Ag/PVA	9	98	Wang *et al.*^91^
5	Ag@TPPQP CMP	8	90	Kotp *et al.*^96^
6	Ag NPs	7	75–98	Karvekar *et al.*^86^
7	Ag@HCMP-bpybph	3	99	Luo *et al.*^47^
8	CS/Ag–LDH film	5	99	Mallakpour *et al.*^88^
9	Chitosan-Ag/Fe_2_O_3_	10	92–95	Batakurki *et al.*^97^
10	Ag NPs	13	98.23	Riaz *et al.*^85^
11	AgNPs/ZnO/Fe_3_O_4_	7	96	Alula *et al.*^78^
12	SiO_2_–Ag@void@SiO_2_ (SAVS)	150	99	Yin *et al.*^93^
13	Ag@SrNbO/LDH	1	97	Zhou *et al.*^94^
14	Ala–Ag NPs	8	95.6	Naaz *et al.*^37^
15	Fe_3_O_4_@PPy-MAA/Ag	45	80	Das *et al.*^76^
16	Fe_3_O_4_/SiO_2_-Pr-S-Ag	2	97	Veisi *et al.*^83^
17	Fe_3_O_4_@TA/Ag	1	99	Veisi *et al.*^84^
18	Ag/N-rGO	1	90	Han *et al.*^90^

a Reaction conditions: NaBH_4_, H_2_O, rt.

#### 2.1.2 Dye degradation

**Fig. 4:**
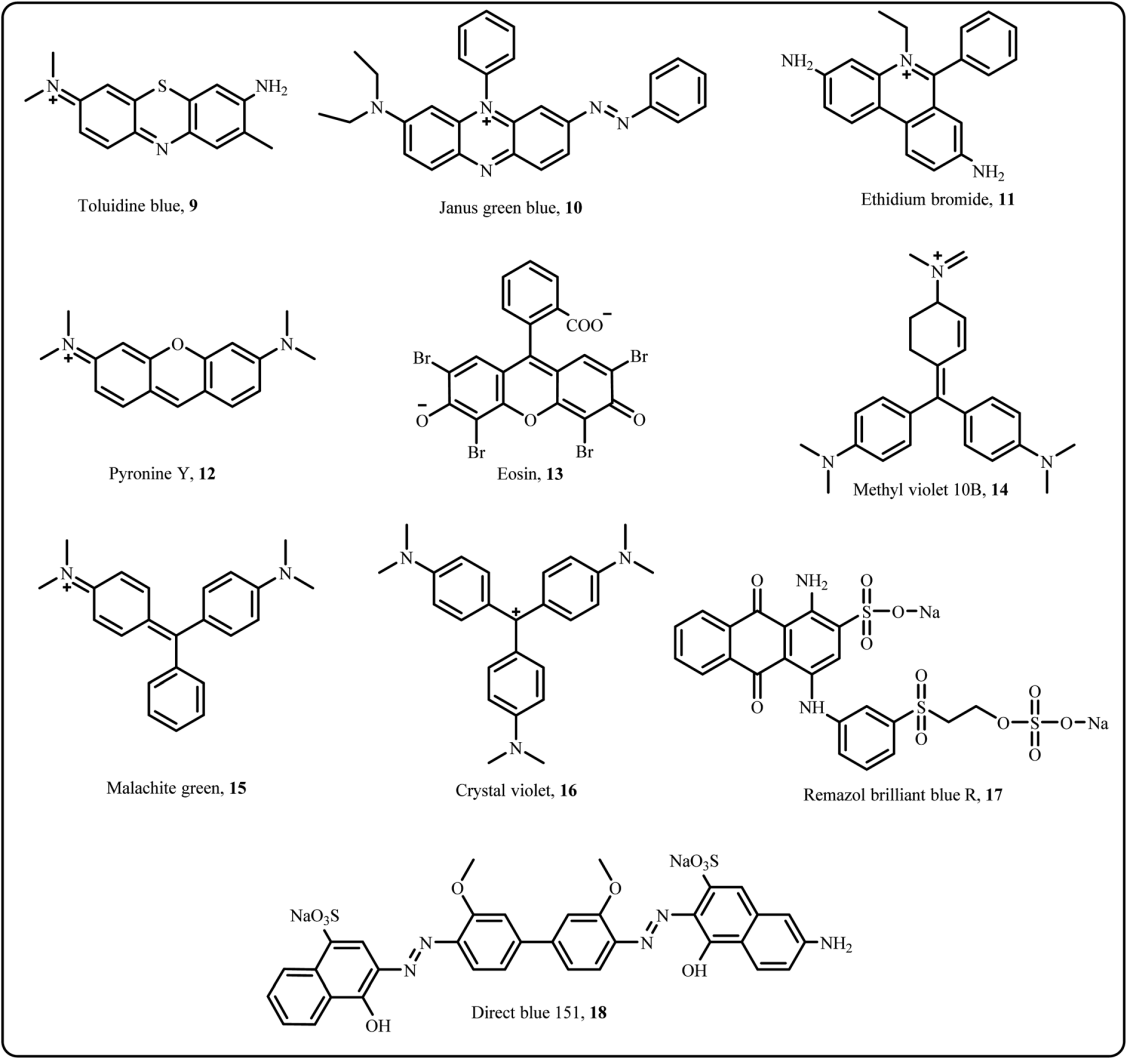
Organic dyes.

Industrial wastewater effluents contain numerous harmful organic dyes, including commonly used ones such as Methylene Blue (MB), Congo Red (CR), Methyl Orange (MO), and Rhodamine B (Rh B), which are prevalent in various industries as colouring agents. Apart from these, other dyes including Toluidine blue, Pyronine Y, Direct blue 151, Eosin and more are widely used across different industrial applications (Fig. 4).^31,48,58,86,98–102^

**Scheme 4:**
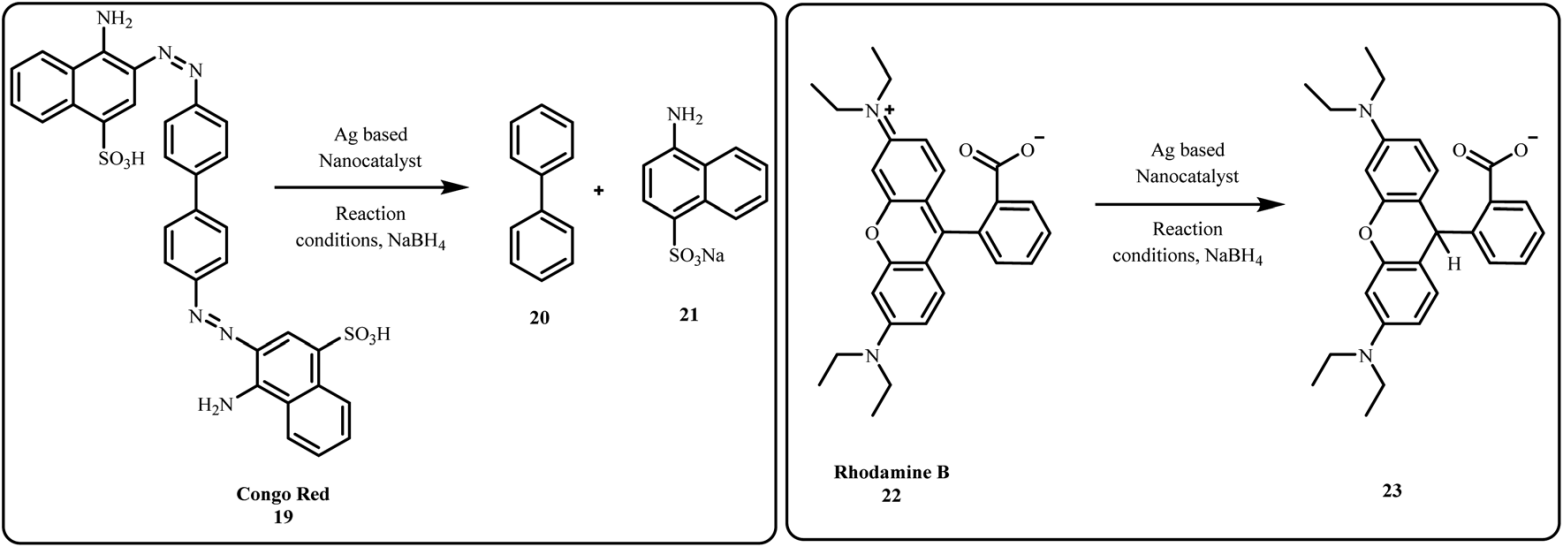
Degradation of CR and Rh B.

**Scheme 5:**
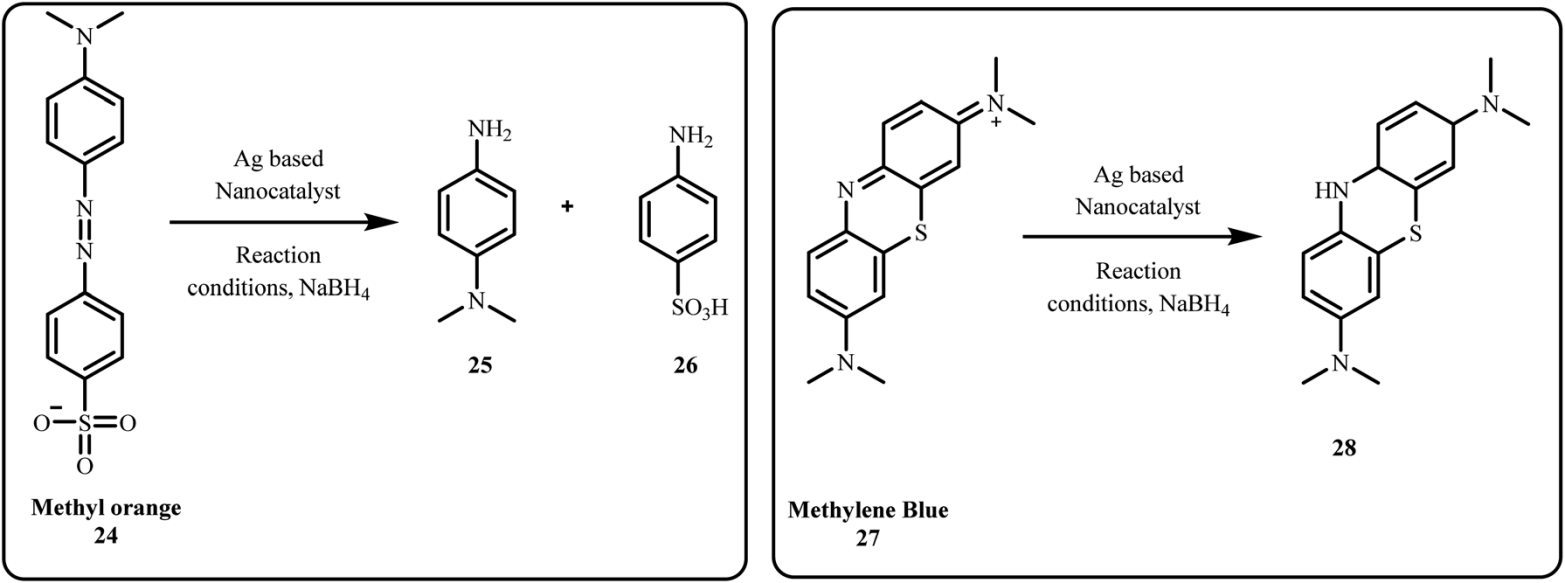
Degradation of MO and MB.

Among these dyes, the catalytic reduction of hazardous azo dyes has recently gained attention from researchers owing to their non-toxic and eco-friendly nature. In particular, various silver-based nanocatalysts such as Ag NPs,^103^ Au/Ag/Fe_3_O_4_@PEI@NC,^104^ GO-Fe_3_O_4_/PAA/Ag,^105^ Ag–MoS_2_,^80^ rGO–Ag NPs,^36^ Fe_3_O_4_@TA/Ag,^84^ Fe_3_O_4_/SiO_2_-Pr-S-Ag,^83^ have been explored for their effectiveness in these catalytic processes (Schemes 4 and 5).

Biogenically produced Ag NPs extracted from mexican mint (MM) leaf extract (MM-AgNPs) successfully catalyzed the decolorization of various dyes namely, Toluidine blue, CR, and Pyronine Y in the presence of NaBH_4_, making it a promising photo nanocatalyst for dye degradation.^31^ Similarly, Thi *et al.* reported that the Ag@AgCl NPs serve as versatile catalysts for the breakdown of azo dyes such as CR by improving the degradation rate by 6–31 times compared to the uncatalyzed reaction.^106^ By employing *Bacillus cereus*, Alfryyan *et al.*, bio-synthesized a pair of novel intracellular and extracellular plasmonic silver-based catalysts *via* a one-pot approach and utilized them for catalytic reduction of MB organic dye.^107^

The study by Farooqi *et al.* aimed to evaluate the catalytic performance of a Ag NPs loaded poly(*N*-isopropylacrylamid-*co*-acrylic acid) [Ag–P(NAA)] microgel system, achieving the complete degradation of malachite green (MG) dye in an aqueous medium in just a few minutes.^48^ The study by Karvekar *et al.* presented an innovative approach combining biogenic synthesis of Ag NPs using *Zingiber officinale* rhizome extract with hydrothermally synthesized ZnO NPs to form Ag–ZnO nanocomposites, which were employed for the photocatalytic degradation of MB and crystal violet under natural sunlight.^86^ A study utilized Ag/ZnO/Fe_3_O_4_ nanocatalyst, synthesized *via* chemical reduction, to catalytically degrade MB demonstrating consistent performance over fifteen consecutive runs without any decline.^78^ In a recent study, Pan *et al.* produced a series of visible light-driven Ag_2_CO_3_/g-C_3_N_4_ nano photocatalysts with varying Ag_2_CO_3_ composition to degrade MO and MB, displaying enhanced photocatalytic efficiencies of 94 and 63% respectively. This improvement, compared to individual Ag_2_CO_3_ or g-C_3_N_4_ catalytic systems, was attributed to the improved dispersion of Ag_2_CO_3_ nanoparticles, reduced particle size and the synergistic effect between Ag_2_CO_3_ and g-C_3_N_4_.^108^

By opting the precipitation method, Khaneghah *et al.* created a series of ternary photocatalysts, consisting of graphitic carbon nitride nanosheets (GCNNS), carbon dots (CD), and Ag_6_Si_2_O_7_ (ASO), denoted as GCNNS/CD/ASO, and assessed the catalytic effectiveness of these catalysts through various reactions. Among the prepared photocatalysts, GCNNS/CD/ASO with 10% ASO content exhibited the optimum performance in degrading Rh B, outperforming other solitary and binary photocatalytic systems.^109^ Thomas *et al.* realized an environment friendly synthesis of Ag NPs by utilising *Myristica fragrans* seed shells extract, an agricultural waste. These NPs were further investigated for the photocatalytic degradation of various dyes such as Rh B (zwitterionic), Remazol brilliant blue reactive (anionic) and methyl violet 10B (cationic), indicating their significant potential for dye degradation.^58^

Green emitting carbon dots were synthesized *via* hydrothermal route, as reported by Ghosh *et al.*, and utilized them to fabricate Ag and Au nanocomposites (CD-AgNP, CD-AuNP). These nanocomposites were explored for degradation of dyes such as CR, MO and Evan's blue (EB), as well as for antibacterial activity.^98^ Recently, chitosan-based silver nanoparticles (Ag NPs) combined with TiO_2_ and ZnO were used to target the photocatalytic degradation of Acid Red 37 dye. The results showed an improvement in dye degradation, with an increase in Ag NP content leading to a faster reaction rate and lower energy consumption.^110^ Similarly, Sodeinde *et al.* prepared reduced graphene oxide–silver (rGO–Ag) nanocomposite *via* green route by using *Corchorus olitorius* extract and waste battery rod powder, achieving 96% photocatalytic degradation of Janus Green Blue (JGB) dye.^99^

Castro *et al.* employed *Anemopsis californica* leaf extract as a reducing agent to fabricate Ag NPs supported on pistachio husk for 100% degradation of a dye containing Direct Blue 151 under natural sunlight.^100^ Similarly, green synthesized Ag NPs were employed for the catalytic destruction of organic dyes such as MB, MO and Rh B, attaining excellent degradation of 96%, 71% and 93% respectively. Additionally, these Ag NPs efficiently worked as a colorimetric sensor for detecting Hg^2+^ and Fe^3+^ ions.^59^ Ag NPs were successfully incorporated by Saruchi *et al.* into a cellulose and gelatin-based hydrogel, C-G-g-poly(AA)-AgNPs, which were employed for the catalytic degradation of carcinogenic dyes such as Ethidium Bromide (EtBr) and eosin.^101^ Eswaran *et al.*, in his recent study, synthesized Ag NPs using *Kalanchoe brasiliensis* extract (KK-AgNPs) and demonstrated their high photocatalytic efficiency in degrading various toxic dyes namely Aniline Blue (86.04%), Toludine Blue (85.95%), CR (78.85%), Indigo Carmine (84.08%), Auramine O (70.40%), and Pyronin Y (66.41%).^102^ Other nanocatalysts listed in Table 2 have also shown effectiveness in degrading the organic dyes (Fig. 4).

**Table 2:** Different dyes degraded by silver-based nanocomposites^a^

S. no.	Catalyst	Time (min)	Dyes degraded	Reference
1	MWCNTs-NH_2_/Fe_3_O_4_/Ag NPs	1, 2	MB, MO	Hamelian *et al.*^38^
2	Fe_3_O_4_@LP-Ag	5, 4, 4	MB, MO, CR	Ahmad *et al.*^80^
3	Au/Ag/Fe_3_O_4_@PEI@NC	6, 14	MB, MO	Iuliano *et al.*^104^
4	RGO–Ag NPs	15, 15	MB, MO	Das *et al.*^87^
5	Ag@AgCl	25	CR	Thi *et al.*^106^
6	rGO–Ag NPs	3	Rh B	Sun *et al.*^36^
7	Extra and intracellular Ag NPs	80, 150	MB	Alfryyan *et al.*^107^
8	Ag–P(NAA)	4	Malachite green	Farooqi *et al.*^48^
9	Ag–ZnO	165	MB	Karvekar *et al.*^86^
10	Ag NPs	45	MO	Riaz *et al.*^85^
11	AgNPs/ZnO/Fe_3_O_4_	10	MB	Alula *et al.*^78^
12	AgNPs@*δ*-FeOOH/PUF	1.3, 3	MO, CR	Mahmoud *et al.*^111^
13	MnFe_2_O_4_@PDA-Ag	4.3	MB	Gürbüz *et al.*^79^
14	GO–Fe_3_O_4_/PAA/Ag	0.5, 2	MB, MO	Esmaili *et al.*^105^
15	Ag-doped MoS_2_ nanopetals	1	MB	Ikram *et al.*^112^
16	Ala–Ag NPs	60, 35	MB, Rh B	Naaz *et al.*^37^
17	κ-CG-*s*-AgNPs	1, 1	MB, Rh B	Pandey *et al.*^113^
18	Fe_3_O_4_/SiO_2_-Pr-S-Ag	0.5, 0.66	MB, Rh B	Veisi *et al.*^83^
19	Fe_3_O_4_@TA/Ag	0.66, 1	MB, Rh B	Veisi *et al.*^84^
20	Ag_2_WO_4_/rGO NCs	120	MB	Malathi *et al.*^53^
21	MMT/Fe_3_O_4_/Ag	8	Rh B	Acar *et al.*^51^
22	VWE-Ag NPs	195, 60	MB, MO	Amjad *et al.*^114^

a Reaction conditions: NaBH_4_, H_2_O, rt.

#### 2.1.3 Other reduction reactions

**Fig. 5:**
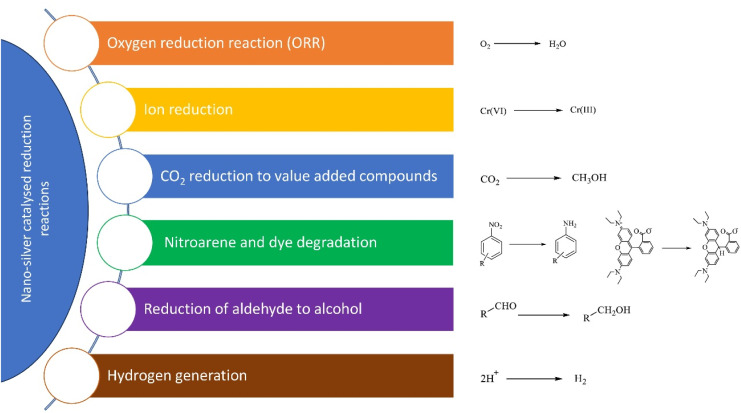
Nanosilver catalyzed various reduction reactions.

Over many decades, Ag-based nanocatalysts have been significantly utilized for realizing numerous reduction reactions (Fig. 5). Qin *et al.* developed a novel supramolecular host motif approach for the electrochemical reduction of CO_2_ to Ethanol through hydroxypillar[5]arene extended porous polymer confined Ag catalytic systems, PAF-PA5-Ag-0.8 and PAF-PA5-Ag-1.9. These systems exhibited improved adsorption of *CO, facilitating ethanol production through C–C coupling, with the former system being the more efficient.^115^ Recently, Ahmad *et al.* developed a novel Ag doped ZnO nanocages and investigated their catalytic hydrogenation of CO_2_ to methanol, a valuable product, using DFT simulations.^62^

**Scheme 6:**
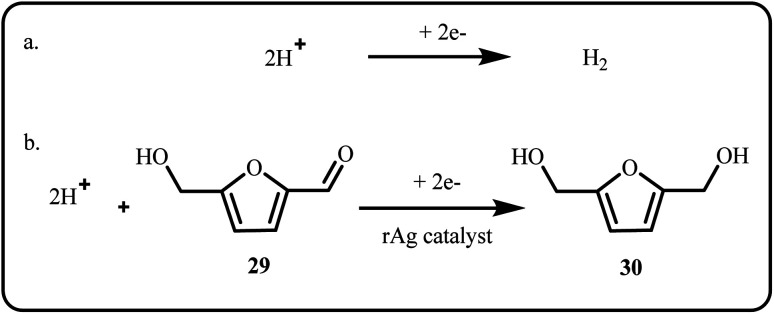
(a) Hydrogen evolution and (b) electrochemical hydrogenation of biomass-derived 5-(hydroxymethyl) furfural (Muchharla *et al.*^32^).

In a separate study, Muchharla *et al.* developed rAg and rCu catalytic systems for the hydrogen evolution and electrochemical hydrogenation of biomass-derived 5-(hydroxymethyl) furfural (HMF) (Scheme 6).^32^

Zhang *et al.* employed a solvothermally synthesized plasmonic Ag/AgCl/NH_2_-UiO-66 for catalytic reduction of Cr(vi) under UV light, where the amine functionalization of UiO-66, along with the hybrid's inorganic–organic nature, significantly enhanced charge separation and transfer efficiency. Additionally, the presence of Ag nanoparticles (Ag NPs) further improved the separation efficiency of photogenerated electrons and holes.^116^ In 2024, numerous research studies have focused on efficient oxygen reduction reaction (ORR) using silver-based nanocatalysts. Nandy *et al.* fabricated a bimetallic [(Ag@AuAg)@Ag] nanocatalyst *via* a template-mediated process for an efficient ORR in fuel cells comparable to traditional Pt/C arrangement, while offering superior stability.^117^ Similarly, an Ag loaded N-doped graphene (Ag-NGs) nanocomposite with high ORR activity was fabricated by Chen *et al.* by opting a simple and environment friendly microwave plasma technique.^118^ Recently, Khaksar *et al.* developed a novel AgVO_3_ blended functionalized multiwalled CNT (AgVO_3_@f-MWCNTs) nanocatalyst that exhibited great ORR catalytic activity.^119^

### 2.2 Oxidation reactions

#### 2.2.1 Oxidation of alcohols

Many researchers have focused on catalyzing alcohol oxidation.^120^ A study by Sobczak *et al.* proposed a one-pot approach for developing a catalyst particularly for the selective oxidation of alcohols while emphasizing on sustainability. The group fabricated silver nanoparticles heavily grafted with stable nitroxide radicals (N–Ag NPs). However, while the N–Ag NPs catalytic system exhibited less satisfactory results with certain alcohols like *n*-heptanol, 1-phenylethanol, and allylic alcohol, it showed high activity and selectivity for primary aromatic alcohols like benzyl alcohol, 4-pyridinemethanol, and furfuryl alcohol, achieving nearly 100% conversion with high yields of the corresponding aldehydes. This efficiency was attained using a reduced amount of catalyst enabling facile purification procedures.^33^ Pham *et al.*^121^ developed a carbon-supported AgPt nano coral through a self-growth-assisted reduction method as a catalytic system. This system demonstrated exceptional HER performance and methanol oxidation in acidic electrolytes, exhibiting considerably lower overpotential and Tafel slope compared to a commercial carbon-supported Pt-based catalyst, highlighting its superior electrocatalytic efficiency.

**Scheme 7:**
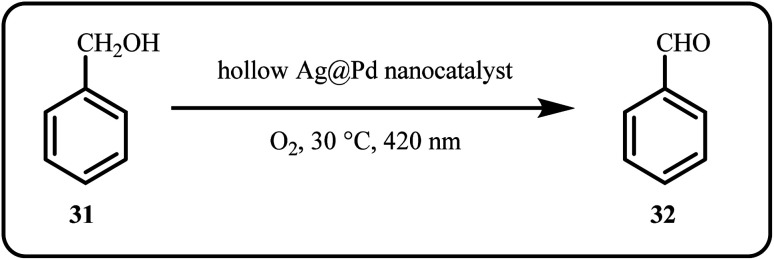
Photocatalytic oxidation of benzyl alcohol using hollow Ag@Pd core–shell nanoparticles (Fang *et al.*^123^).

Additionally, the catalyst exhibited an improved reaction rate and resistance to CO poisoning during methanol oxidation. This enhanced performance resulted from the synergistic and electronic interactions between Ag and Pt.^121^ A novel nano-catalyst, consisting of zeolite, carbon nanotubes (CNT), and silver nanoparticles, was developed for the electrochemical oxidation of propylene glycol (PG) in sulfuric acid solution. The group observed a significant enhancement in the electrocatalytic activity toward propylene glycol oxidation with increasing CNT content, as monitored through techniques like cyclic voltammetry, chronoamperometry, and electrochemical impedance spectroscopy.^122^ Fang *et al.* proposed a plasmon-mediated approach to fabricate novel hollow Ag@Pd core–shell nanoparticles, which efficiently catalyzed the oxidation of benzyl alcohols to aldehydes under visible light illumination (Scheme 7).^123^

**Scheme 8:**
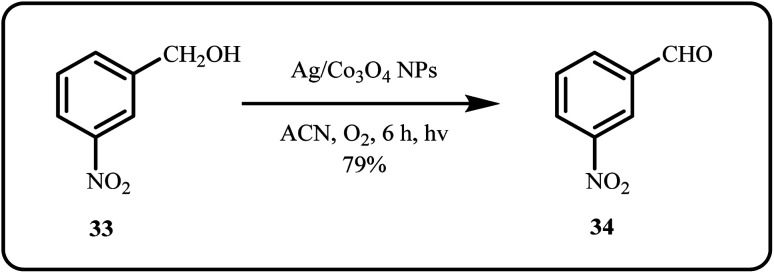
Photocatalytic oxidation of aromatic alcohols using Ag/Co_3_O_4_ nanoparticles (Ji *et al.*^124^).

An *in situ* prepared silver supported on cobalt oxide (Ag/Co_3_O_4_) nanocatalyst was applied for the one-pot photocatalytic oxidation of aromatic alcohols (Scheme 8), where 1 wt% of silver-loaded catalyst exhibited optimal performance, showing over 99% selectivity with a conversion rate of 76% toward the desired aldehyde product.^124^

**Scheme 9:**
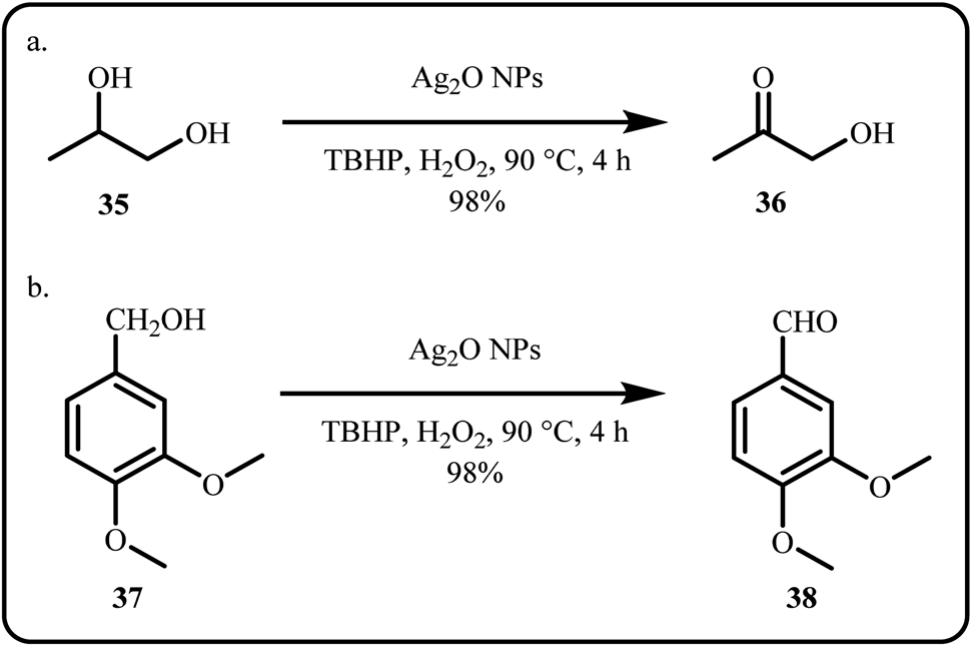
Oxidation of (a) propylene gylcol and (b) veratryl acohol (Devanathan *et al.*^126^).

For ethanol electrooxidation, Pawar *et al.* recently developed a Pd@Ag–Zn–rGO catalytic system that exhibited seven times higher catalytic activity than the conventional Pd/C system and outperformed other existing Pd-based systems due to the synergistic interactions between Pd and alloy support.^125^ Recently, Devanathan *et al.* proposed silver oxide nanoparticles (Ag_2_O NPs) for the selective oxidation of propylene glycol and veratryl alcohol (Scheme 9).^126^

Hatshan *et al.* catalytically oxidised a wide array of aromatic, heterocyclic, allylic, primary, secondary, and aliphatic alcohols to their respective ketones and aldehydes using Ag_2_O–MnO_2_/(*X*%)N-DG, with N-DG/MnO_2_-(1% Ag_2_O) displaying premium activity with 100% conversion and >99.9% selectivity.^127^

#### 2.2.2 Other oxidation reactions

Khani *et al.* comparatively studied citric-acid-modified polyaniline (P-CA) and its Ag nanoparticle-modified counterpart (Ag@P-CA) with unmodified polyaniline (PANI) and PANI-modified Ag nanoparticles (Ag@PANI) for the electrochemical oxidation of nitrophenol, where citric acid surface modification improved the monolayer adsorption capacity by reducing the binding affinity while enhancing the conductivity of P-CA and Ag@P-CA, thereby enabling more efficient electrochemical nitrophenol reduction–oxidation.^128^

In a recent work, morin, an organic dye, was catalytically degraded with quercetagetin-stabilized Ag NPs (Que-AgNPs) by oxidative means using H_2_O_2_, following pseudo-first-order rate kinetics. Furthermore, the cytotoxicity studies were conducted against HeLa and MCF-7 cell lines where Que-AgNPs exhibited dose-dependent cytotoxic activity.^129^ Similarly, Yilmaz *et al.* opted hyaluronic acid-functionalized Ag NPs (HA-AgNPs) for the catalytic oxidative destruction of morin dye with H_2_O_2_.^130^

**Scheme 10:**
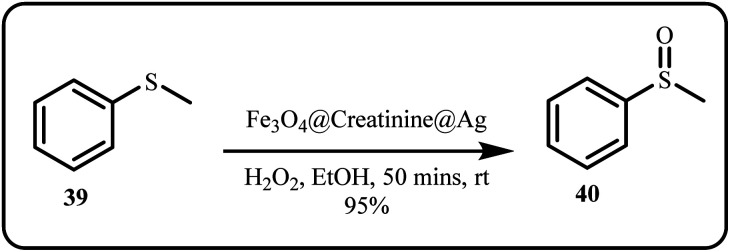
Oxidation of sulfides catalyzed by Fe_3_O_4_@Creatinine@Ag (Molaei *et al.*^42^).

Molaei *et al.* highlighted the synthesis of a recoverable recyclable magnetic nanocatalyst, Fe_3_O_4_@Creatinine@Ag, prepared *via* functionalizing the surface of Fe_3_O_4_ nanoparticles with creatinine attached to Ag NPs. Subsequently, this catalytic system was applied to selectively oxidize sulfides (Scheme 10) and synthesize 5-substituted 1*H*-tetrazoles where the catalyst, affording excellent yields of the corresponding sulfoxides without any harsh reaction conditions.^42^

**Scheme 11:**
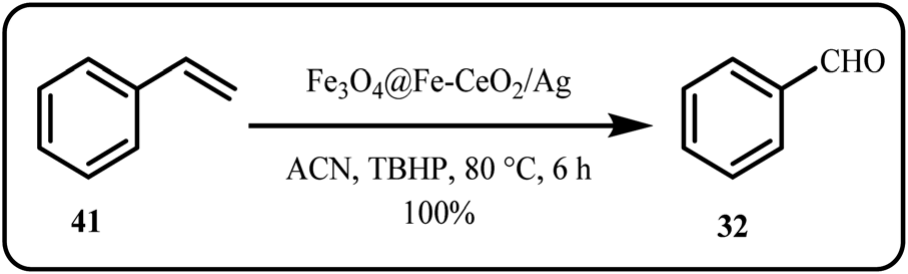
Oxidation of styrene catalyzed by Fe_3_O_4_@Fe–CeO_2_/Ag (Ghosh *et al.*^131^).

Recently, Zhang *et al.* employed a tandem catalyst, Ag/CeSnO_*x*_, as an efficient catalyst to selectively oxidize low-concentration NH_3_ from exhaust gases to N_2_.^35^ Ghosh *et al.* fabricated a novel ternary composite nanocatalyst, composed of an Ag-decorated Fe_3_O_4_ core, Fe-doped CeO_2_ shell, and Ag NPs (Fe_3_O_4_@Fe–CeO_2_/Ag), which was employed for the selective oxidation of various alkenes, including styrene, achieving 100% conversion and 95% selectivity for benzaldehyde (Scheme 11). In addition, the catalytic system exhibited remarkable stability over 4 cycles and minimal loss in activity.^131^

**Scheme 12:**
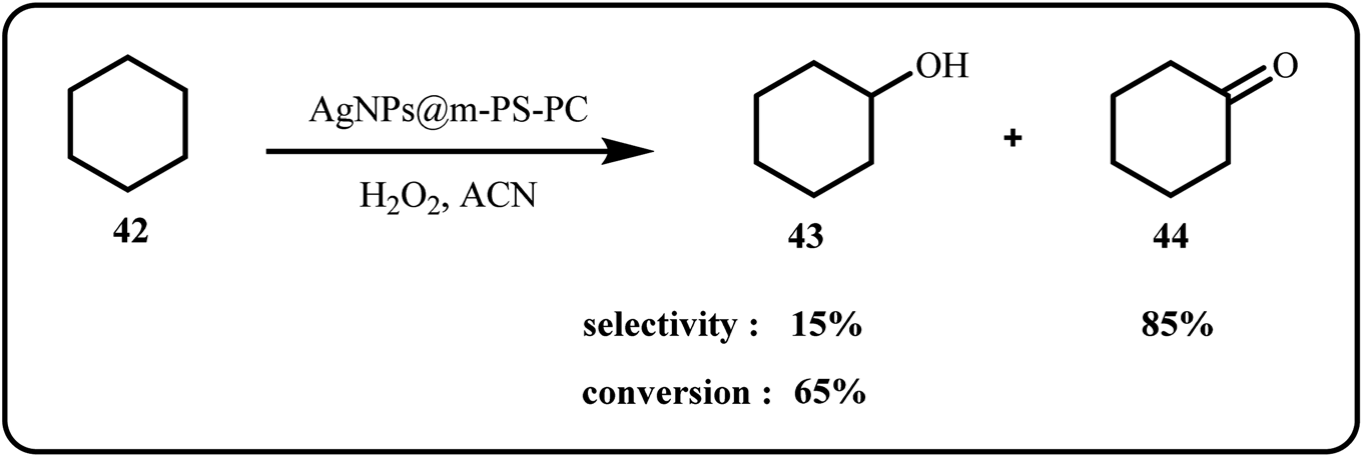
Oxidation of cyclohexane catalyzed by AgNPs@m-PS-PC nanocomposite (Salam *et al.*^40^).

Bahadorikhalili *et al.* employed ultrasonic synthesis to successfully develop a novel nanocatalyst, Ag@mHAp-Si-(S) by confining Ag NPs over thiourea-functionalized magnetic hydroxyapatite support. Later on, the group applied this catalytic system to effectively oxidize primary amines using urea hydrogen peroxide (UHP).^46^ Dey *et al.*, fabricated Cu@Ag/MWCNT nanocomposite that demonstrated extraordinary electrocatalytic performance for the oxidation of borohydride. Moreover, the synthesized catalyst outperformed the carbon supported Cu@Ag (Cu@Ag/C) system by 3.8 times.^132^ In a novel approach, Salam *et al.* designed a nanocatalyst, Ag NPs@m-PS-PC, consisting of Ag NPs, polystyrene amine and 2-pyridinecarbaldehyde. The catalytic activity was evaluated *via* cyclohexane oxidation using hydrogen peroxide (Scheme 12), affording high activity and selectivity as well as green synthesis of carboxylic acids through CO_2_ fixation under ambient conditions.^40^

**Scheme 13:**
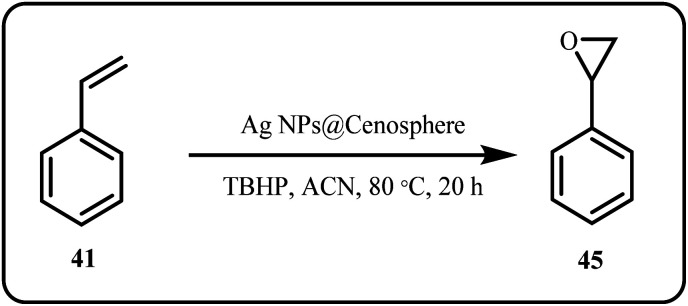
Epoxidation of styrene catalyzed by AgNPs@Cenosphere (Tarasova *et al.*^134^).

Pugazhenthiran *et al.* employed Ag NPs loaded ZnO nanostructures (Ag-ZnONSTs) for the photocatalytic degradation of ceftiofur sodium (CFS), where Ag NPs loading contributed to the enhanced photocatalytic activity of Ag-ZnONSTs compared to TiO_2_ (P25) NPs, with Ag-ZnONRs exhibiting the highest oxidation rate constant (*k*′ = 4.6 × 10^−4^ s^−1^).^133^ In 2024, Tarasova *et al.* utilized waste fly-ash cenosphere as a support for Ag nanoparticles, developing a heterogenous catalysts for the epoxidation of styrene (Scheme 13) to produce value added products in high yields.^134^

### 2.3 Coupling reactions

Herein, we will review the nanosilver catalyzed Suzuki and A^3^ coupling reaction. The Suzuki coupling is a model reaction in forming carbon–carbon bonds with tolerance to various functional groups, such as esters, ketones, and nitriles to produce a variety of complex molecules having widespread applications in pharmaceuticals, natural products, polymers *etc.*^34^ The A^3^ coupling is a fascinating multicomponent reaction involving the simultaneous coupling of an aldehyde, an alkyne, and an amine, to provide proparglyamines, a class of compounds diversely exploited in the fields of pharmaceuticals, materials science, and beyond.^29^

#### 2.3.1 Suzuki coupling

**Scheme 14:**
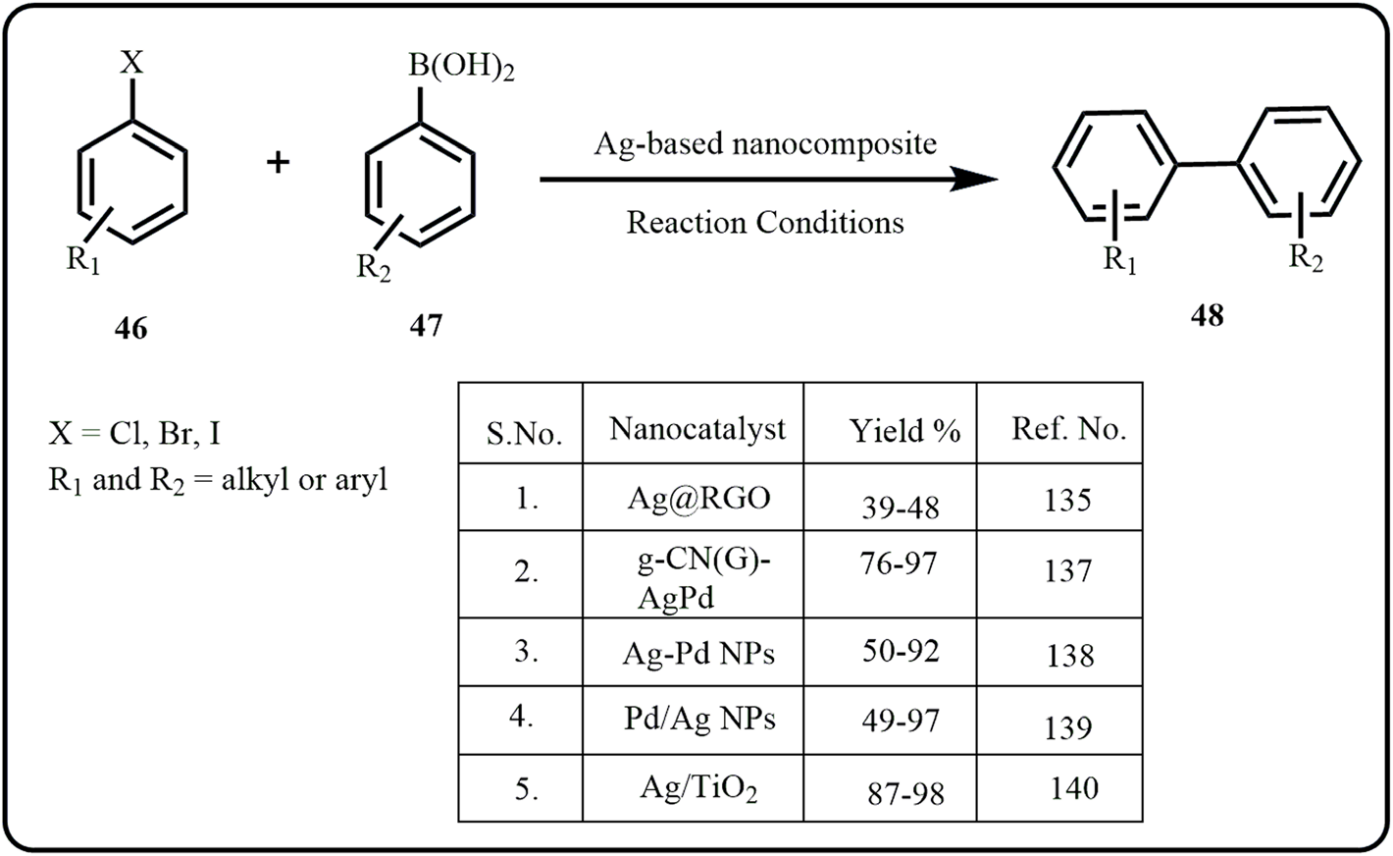
Synthesis of biaryls *via* Suzuki coupling.

Traditionally, the process involved coupling of aryl or vinyl halides with boronic acids or boronate esters under the catalytic influence of palladium. However, challenges such as difficult separation and purification, toxicity and non-reusability of catalysts have enabled researchers to explore Pd-free alternatives without affecting the yield and quality of the obtained products. Herein, we have accounted for numerous research works employing highly efficient recyclable silver-based nanocatalyst in Suzuki–Miyaura cross coupling reaction (Scheme 14). Interestingly, most of these prepared nanocatalysts were reused for 3 to 7 consecutive runs without compromising the yield and quality of the product.^135^

Recently, Ameen *et al.* fabricated metal/metal oxide doped reduced graphene oxide nanocomposites (Ag@RGO, Au@RGO, ZrO_2_@RGO) *via* green routes as a Pd-free catalytic system for Suzuki cross coupling reaction to successfully obtain various biphenyls affording product yield comparable to the conventional palladium-based catalyst, with Ag@RGO offering the highest yield.^135^ Nasr *et al.* reported that Ag, Cu, and Au NPs could be incorporated into clay (K-10) supports (SNPS-K-10, GNPS-K-10, and CNPS-K-10) to give clay-based metal nanocatalyst for Suzuki–Miynaura C–C coupling. Interestingly, the group produced numerous biphenyls *via* two synthesis routes-heating and visible light irradiation, wherein significant yields were obtained through visible light irradiation compared with the traditional heating route.^136^ Altan *et al.* developed g-CN(G)-AgPd and investigated the effect of band bending over the photocatalytic coupling reaction, that offered excellent yields. The depletion layer width influenced the Schottky barrier properties of the produced nanocomposite, resulting in their exceptional photocatalytic performance.^137^

**Scheme 15:**
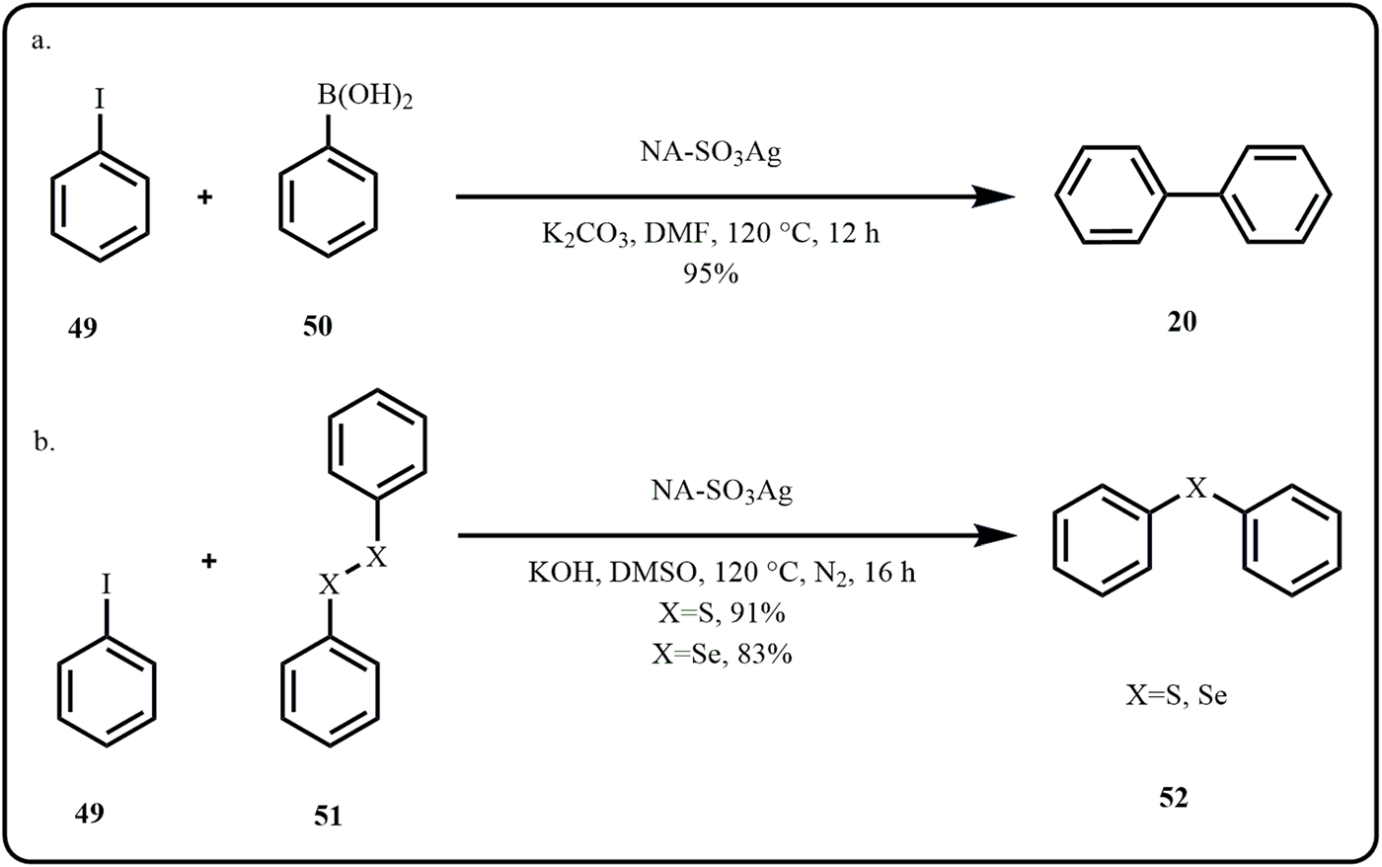
(a) C–C, (b) C–S and C–Se coupling reactions catalyzed by NA-SO_3_Ag (Zolfaghari *et al.*^141^).

In a report by Shaikh *et al.*, bimetallic Ag–Pd nanoparticles supported on SBA were used for the Suzuki coupling reaction, with natural waste rice husk ash silica serving as a support for the ionic liquid and nanoparticle system, preventing aggregation, while also demonstrating enhanced catalytic efficiency of the Ag–Pd system over the monometallic counterpart due to synergistic interaction.^138^ Fascinated by bimetallic nanoparticle catalysts and metal-reducing bacteria, Kimber *et al.*, highlighted the one-step biosynthesis of two bimetallic nanoparticle catalytic systems, Pd/Ag and Pd/Au, that demonstrated superior catalytic performance in Suzuki–Miyaura cross-coupling reaction compared to monometallic Pd catalytic system.^139^ Chen *et al.* discussed the visible-light assisted C–C coupling using an *in situ* prepared Ag/TiO_2_ nano photocatalyst. Interestingly, the nanocomposite exhibited enhanced catalytic efficiency emerging from the synergistic interactions between silver and titania.^140^ A novel mineral carbonaceous silver-based nanocatalyst, NA-SO_3_Ag, was developed *via* grafting techniques to facilitate C–C, C–S, C–Se coupling reactions to offer excellent yields of various biphenyls, aromatic sulfides, and selenides (Scheme 15).^141^

**Scheme 16:**
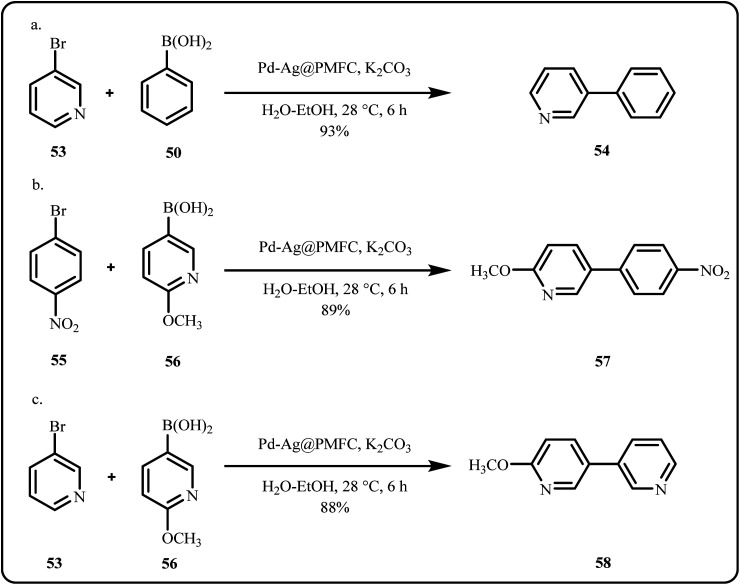
Bimetallic catalyzed Suzuki–Miyaura coupling (a)–(c) of nitrogen rich heterocycles (Bhattacharjee *et al.*^142^).

Recently, Bhattacharjee *et al.* employed waste pomegranate peels to fabricate biocompatible cellulose fibers as an excellent support matrix for bimetallic Pd–Ag nanoclusters (Pd–Ag@PMFC) for catalytic Suzuki–Miyaura coupling of nitrogen-rich heterocycles under visible light conditions (Scheme 16), achieving great results emerging from the synergistic interactions between bimetallic centers.^142^

#### 2.3.2 A^3^ coupling

**Scheme 17:**
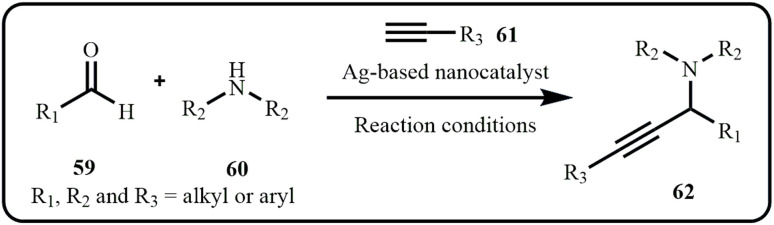
Synthesis of propargylamines *via* A^3^ coupling.

The A^3^ coupling reaction is an important multicomponent reaction for synthesizing propargylamines (Scheme 17), which serve as crucial precursors for a wide range of significant heterocyclic compounds, including pyrroles, pyridines, oxazoles, and others. These derivatives are pivotal in the synthesis of numerous biologically active molecules, such as β-lactams, conformationally restricted peptides, and isosteres, as well as in the development of fungicides, herbicides, and therapeutic drugs. Moreover, propargylamines exhibit diverse biological activities, including anticancer, antimicrobial, and enzyme inhibitory properties. Driven by this, researchers worldwide are focusing on the efficient synthesis of propargylamine. In this context, we present the latest silver nanocatalytic methods for synthesizing propargylamine. Most of the catalytic systems maintained remarkable reusability for 3 to 10 cycles with minimal loss in activity.^39^

In another study, Aparna *et al.* fabricated silver nanoparticles and integrated them into a metal–organic framework (Ag@UiO-66-SH) that was defectively thiol-functionalized by Zr. The synergistic silver–sulfur interactions enhanced the catalytic activity, leading to significant yields in propargylamine synthesis.^143^

Recently, Xie *et al.* fabricated a Cu^2+^-based MOF and loaded with Ag NPs into the pores to generate an efficient nanocatalyst for the A^3^-coupling reaction.^144^ Wang *et al.* efficiently synthesized diverse propargyl amines (70–98%) by utilizing a novel nanocatalytic system prepared by incorporating Ag NPs on biguanide-modified mesoporous silica KIT-5 (KIT-5-bigua-Ag). Furthermore, they explored the biological activity of the material through antioxidant and anticancer assays (IC_50_ = 915.22l g mL^−1^ against the A549 cell line), making it chemotherapeutic.^39^

Interestingly, Mariconda *et al.* evaluated and compared the efficiency of four novel synthesized NHC-based silver and gold catalysts in A^3^ coupling reaction. Herein, the group concluded that the catalytic activity depended on both metal as well as the NHC backbone.^145^

Likewise, Dou *et al.* combined Ag-complexes with Keggin polyoxometalate (POM) to fabricate two novel inorganic–organic Ag-POM hybrids, featuring three-coordinated and distorted tetrahedral configurations, respectively.^146^ Driven by an interest in magnetically isolable nano-biocomposites, Ma *et al.* designed and developed a novel core–shell magnetic nanocomposite, Ag/Bigua-CS@Fe_3_O_4_, by integrating silver nanoparticles onto a biguanidine-chitosan (Bigua-CS) dual biomolecular-functionalized coating. They investigated its catalytic performance for one-pot A^3^ coupling in water, achieving excellent yields of propargylamine derivatives.^147^

Recently, Rafiee *et al.* concentrated a study involving the synthesis of a novel magnetic bio-nanocatalyst, Fe_3_O_4_@CS-StOX@Cys@Ag^+^, composed of chitosan cross-linked with starch oxide, functionalized with cysteine, and immobilized with Ag ions. The catalytic system was then investigated for its performance *via* one-pot three-component A^3^ coupling reaction, achieving great yields of propargylamine derivatives while offering numerous advantages such as the absence of base, cocatalyst, or side reactions.^45^ Similarly, Veisi *et al.* described the robust bio-synthesis of Ag NPs utilizing orange peel extract, that were catalytically explored *via* three-component A^3^ coupling affording excellent results.^153^Table 3, displays a comparative study of various nanosilver catalysts used for A^3^ coupling.

**Table 3:** A comparative study of A^3^ coupling catalyzed by Ag nanocomposites

S. no.	Catalyst	Reaction condition	Time (h)	Yield (%)	Reference
1	Ag MNPs	H_2_O, 60 °C	0.5	96	Babaei *et al.*^148^
2	g-C_3_N_4_-TCT-2AEDSEA-Ag-Cu-Ni	Toluene, 80 °C	8	90	Zarei *et al.*^149^
3	Ag@UiO-66-SH	ACN, 80 °C	6	98	Aparna *et al.*^143^
4	Ag-NPs@PDVTA-1	Neat, 100 °C	12	83	Chandra *et al.*^150^
5	KIT-5-bigua-Ag	H_2_O, 80 °C	8	98	Wang *et al.*^39^
6	Fe_3_O_4_-g-C_3_N_4_-Alg-Ag	H_2_O, rt	0.25	95	Daraie *et al.*^151^
7	AgNPs/Fe_3_O_4_@chitosan/PVA	EtOH, ultrasonic 40 °C	0.3	98	Ghasemi *et al.*^152^
8	CNT-Fe_3_O_4_-fibroin-Ag	Neat, 80 °C	2	92	Akbarzadeh *et al.*^29^
9	Ag-POMs	CH_3_CN, rt	6	99	Dou *et al.*^146^
10	Ag/Bigua-CS@Fe_3_O_4_ NPs	H_2_O, 80 °C	10	96	Ma *et al.*^147^
11	Fe_3_O_4_@CS-StOX@Cys@Ag^+^	EtOH, reflux or toluene, 110 °C	0.5 or 1.3	97 or 95	Rafiee *et al.*^45^
12	Ag/EOs orange NPs	Toluene, 80 °C	8	95	Veisi *et al.*^153^
13	Fe_3_O_4_@R tinctorum/Ag NPs	H_2_O, 80 °C	8	96	Veisi *et al.*^154^

### 2.4 Click reactions

#### 2.4.1 Triazole synthesis

**Scheme 18:**
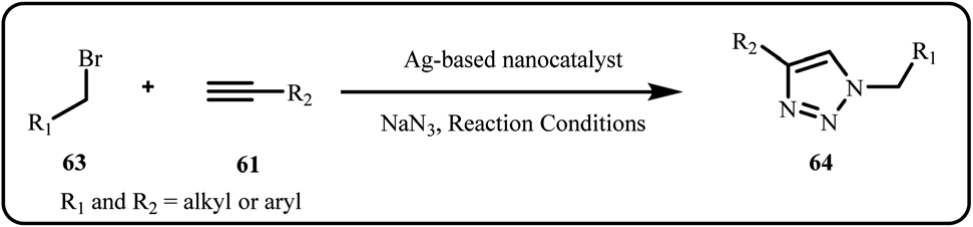
Synthesis of triazole synthesis *via* Click mechanism.

The Click reaction, specifically the Huisgen azide–alkyne cycloaddition (AAC), is a crucial reaction particularly for the one-pot synthesis of triazoles (Scheme 18). Herein, an azide and alkyne undergo [3 + 2] cycloaddition giving a high yield regioselective 1,2,3-triazole moiety. Triazoles serve as versatile organic compounds finding applications in medicinal chemistry, materials science, polymer chemistry and many more. Conventionally, the reaction requires copper-catalysts, however, many recent researches highlight the use of silver-based catalysts to synthesize triazoles with improved results.^152^

**Scheme 19:**
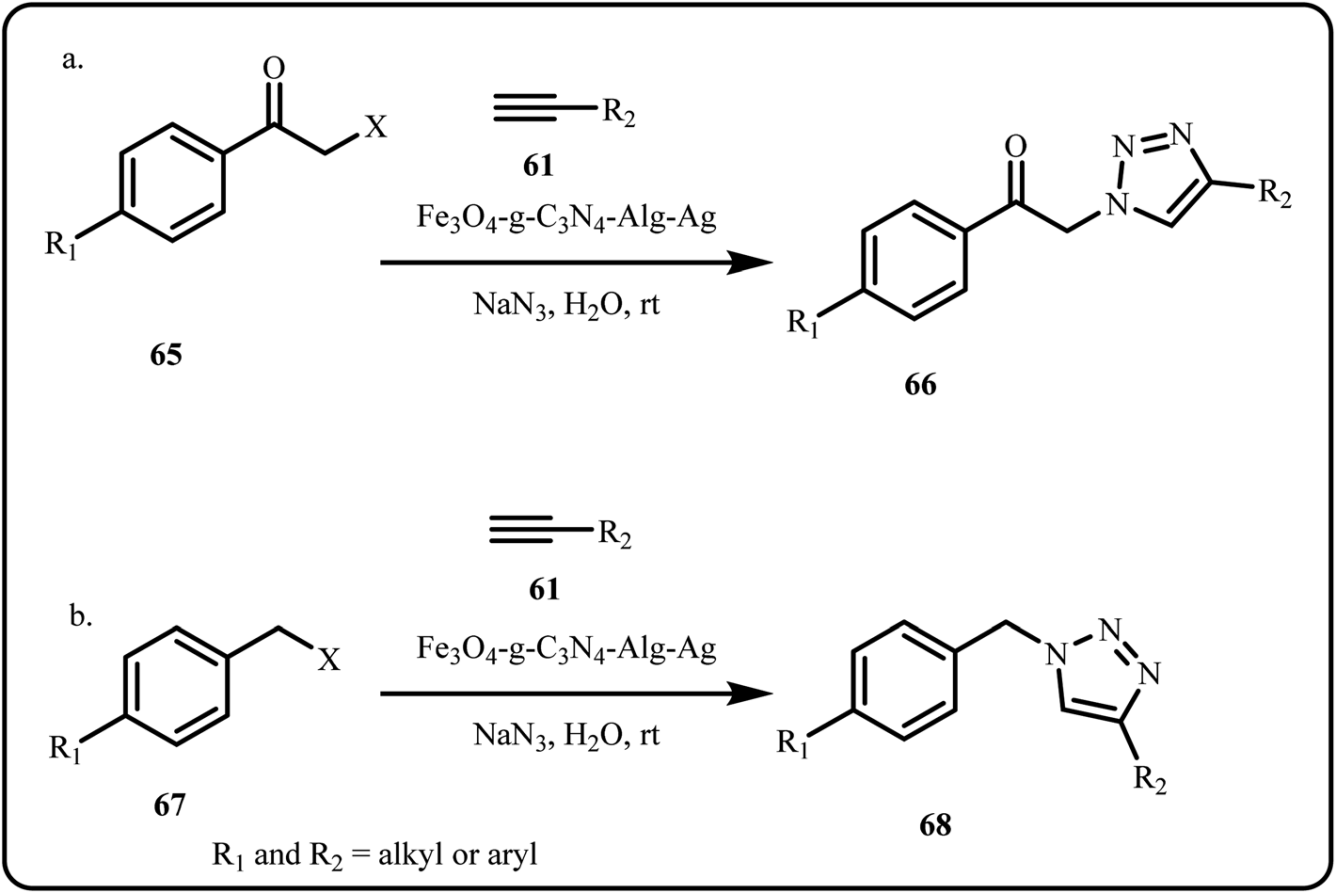
Synthesis of 1,4-disubstituted 1,2,3-triazoles from (a) α-haloketone and (b) alkyl halide using Fe_3_O_4_/g-C_3_N_4_/Alg-Ag catalytic system (Daraie *et al.*^151^).

A novel and efficient nanocatalyst, Fe_3_O_4_/g-C_3_N_4_/Alginate-Ag, was successfully synthesized and demonstrated promising catalytic activity in the regioselective synthesis of 1,4-disubstituted 1,2,3-triazoles *via* a click reaction in aqueous media (Scheme 19). This catalyst provided shorter reaction times, higher efficiency, and improved product purity.^151^

Darroudi *et al.* designed and developed a hydrophilic benzimidazole functionalized fumed silica-based fluorescent nanocomposite, highly sensitive and selective to Ag^+^ ions. Subsequently, Ag(i) was successfully incorporated, producing Ag(i)@Fum-Pr-Pyr-benzimidazole nanocatalyst that catalytically synthesized 1,4-disubstituted triazoles through a green route.^155^ A novel eco-friendly magnetic nanocatalyst, AgNPs/Fe_3_O_4_ @chitosan/PVA was fabricated for the quick one-pot synthesis of triazole derivatives, by functionalizing chitosan bio-polymeric chains with PVA, followed by the addition of Ag NPs and Fe_3_O_4_.^152^

#### 2.4.2 Tetrazole synthesis

**Scheme 20:**
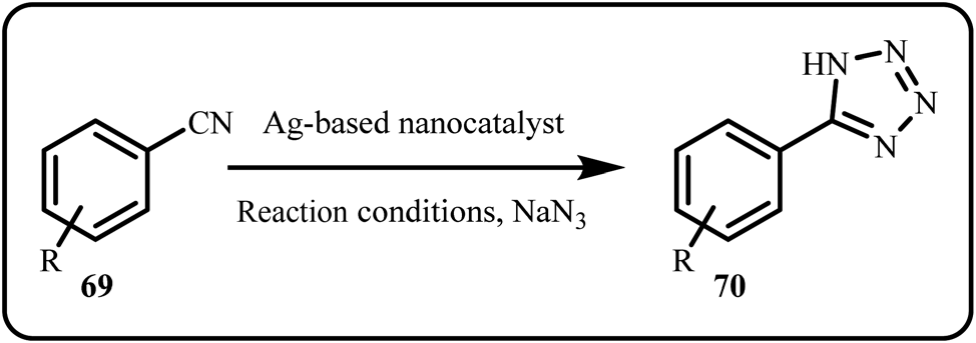
Tetrazole synthesis *via* Click reaction.

The Click reaction, adapted for tetrazole synthesis (Scheme 20), represents a pivotal reaction for constructing heterocyclic scaffolds in organic chemistry. Typically, tetrazole synthesis involves the azide-nitrile cycloaddition under low pH conditions, with or without a catalyst, depending on the reaction conditions and substrate compatibility.^42^

**Scheme 21:**
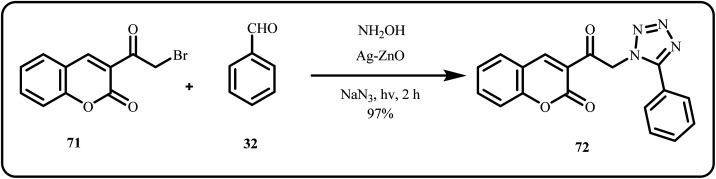
Photo assisted synthesis of novel (1*H*-tetrazol-5-yl)-coumarin hybrids (Attia *et al.*^158^).

A novel material, CF/MC/HA/A, was fabricated by Molaei *et al.*, offering an innovative approach for the catalytic synthesis of 5-substituted 1*H*-tetrazoles *via* green routes. The core–shell structure was attained by modifying Mobil Composition of Matter (MCM-41) NPs with 3,4,5-trihydroxyphenyl acetic acid (HA) and Ag(i) over a mesoporous ferromagnetic CoFe_2_O_4_ (CF) spinel. This innovative strategy provided an effective, reusable catalysts with easy recovery and eliminating the use of hazardous catalysts in an environment-friendly method.^156^ Prakash *et al.* introduced biocompatible gum acacia modified Ag–TiO_2_ and Ag–SiO_2_ nanostructures as heterogeneous catalysts for *in situ* synthesis of 5-substitued 1 *H*-tetrazoles *via* [3 + 2] cycloaddition using aryl nitriles and sodium azide.^157^ Molaei *et al.* functionalized the Fe_3_O_4_ surface with 3-chloropropyltrimethoxysilane, to bind with creatinine, followed by confinement of Ag resulting in Fe_3_O_4_@Creatinine@Ag nanocatalyst. The proposed catalyst system successfully synthesized 5-substituted 1*H*-tetrazoles under mild and eco-friendly conditions.^42^ Novel tetrazole-coumarin hybrids were prepared photocatalytically using an Ag/ZnO nanocatalyst and ZnO nanorods *via* a one-pot facile route, affording excellent product yield (Scheme 21). Moreover, some of the synthesized tetrazole derivatives displayed anticancer activity against MCF-7, HepG2, A549 and Wi38 cancer cell lines.^158^

**Scheme 22:**
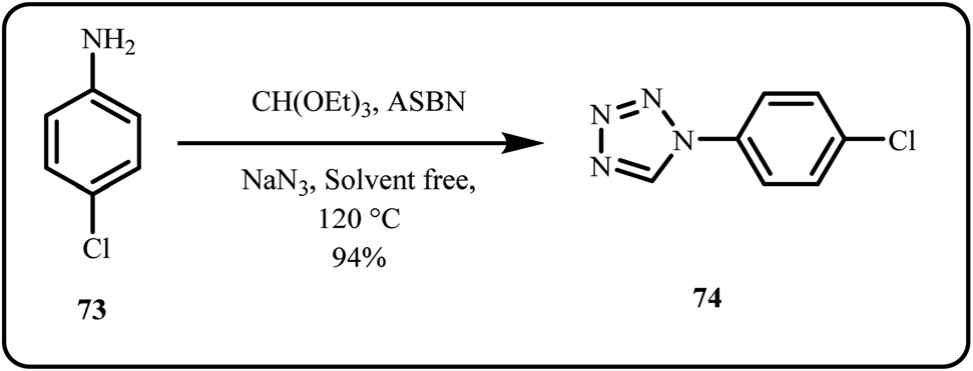
Synthesis of 1*H*-1,2,3,4-tetrazoles using the ASBN catalyst (Nasrollahdeh *et al.*^159^).

Nasrollahzadeh *et al.* utilized *A. moluccana* extract to synthesize a nanocatalyst comprising of sodium borosilicate glass-supported silver NPs (ASBN) *via* a green route. Herein, this catalytic system was applied for solvent-free [3 + 2] cycloaddition of amines with sodium azide, yielding assorted 1-substituted-1,2,3,4-tetrazoles in a cost-effective and environmental-friendly manner (Scheme 22). Furthermore, protein binding and toxicology studies were conducted to determine the ecological impact.^159^

### 2.5 Miscellaneous reactions

**Scheme 23:**
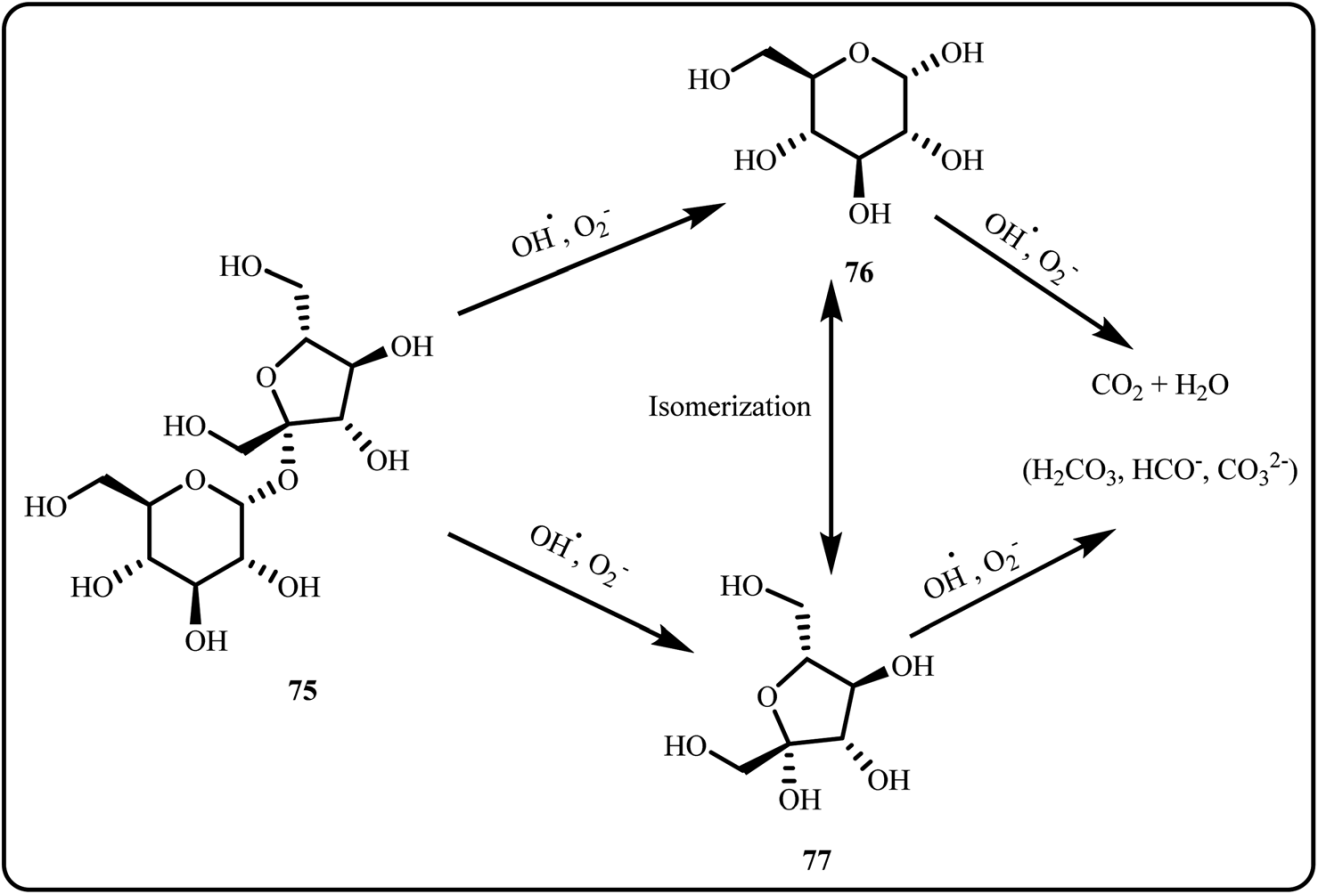
Photocatalytic degradation of sucrose using Ag/ZnO (Buengkitcharoen *et al.*^160^).

A ZnO and Ag nanoparticle nanocomposite (Ag/ZnO) was synthesized *via* a two-step method, starting with sol–gel autocombustion followed by ion-impregnation. The incorporation of Ag NPs onto the ZnO surface reduced the electron–hole pair recombination by enabling direct electron transfer to metallic Ag, facilitating better photocatalytic activity. The mechanism involved hydroxyl radicals, to catalytically photodegrade the sucrose into glucose and fructose, eventually leading to its complete conversion into CO_2_ and H_2_O (Scheme 23). The 10% Ag/ZnO catalytic system exhibited exceptional condensate degradation up to 90% within 90 minutes.^160^

**Scheme 24:**
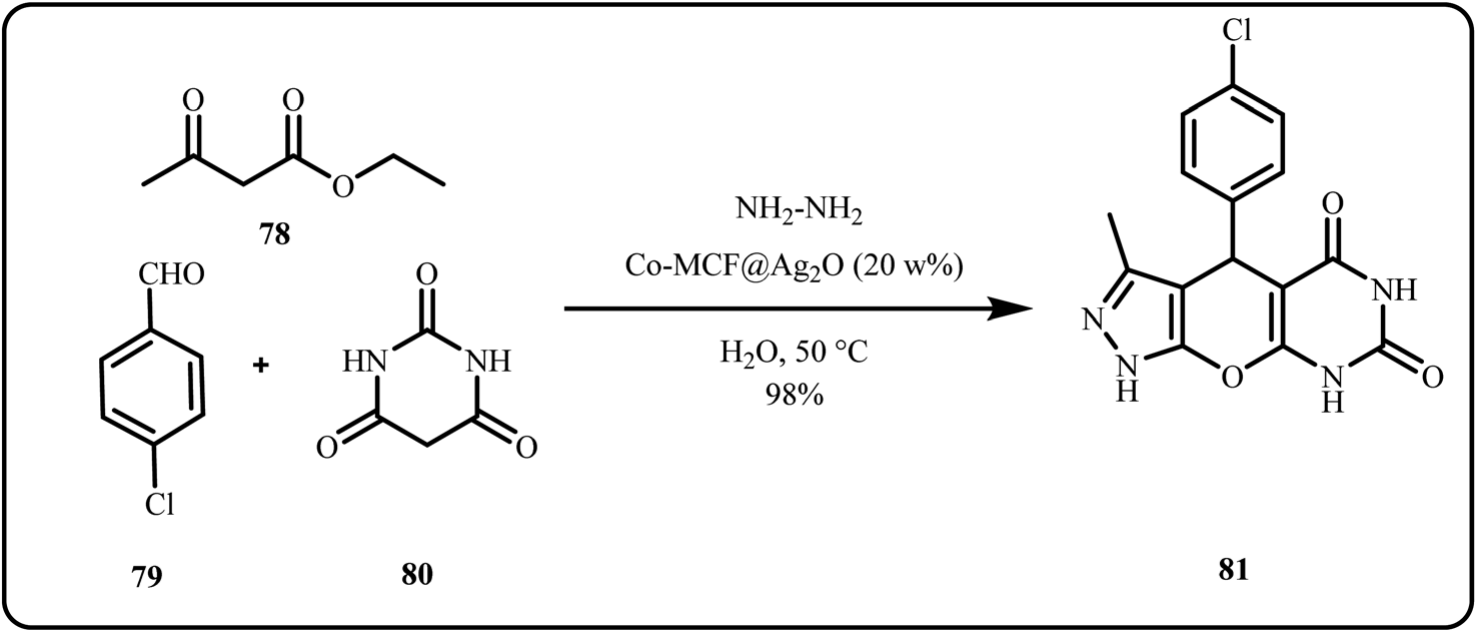
One-pot synthesis of pyrazolopyranopyrimidines catalyzed by Co-MOF-Ag_2_O (Hootifard *et al.*^161^).

Hootifard *et al.* opted co-precipitation procedure followed by microwave irradiation to produce a Co-MOF stabilized Ag_2_O nanocomposite (Co-MOF@Ag_2_O) and employed it for the one-pot synthesis of various tricyclic fused pyrazolopyranopyrimidines in water at 50 °C, achieving high yields in short reaction times (Scheme 24). The study highlights cost-effectiveness, catalyst recyclability, and an environmentally friendly, solvent free approach as key advantages.^161^

**Scheme 25:**
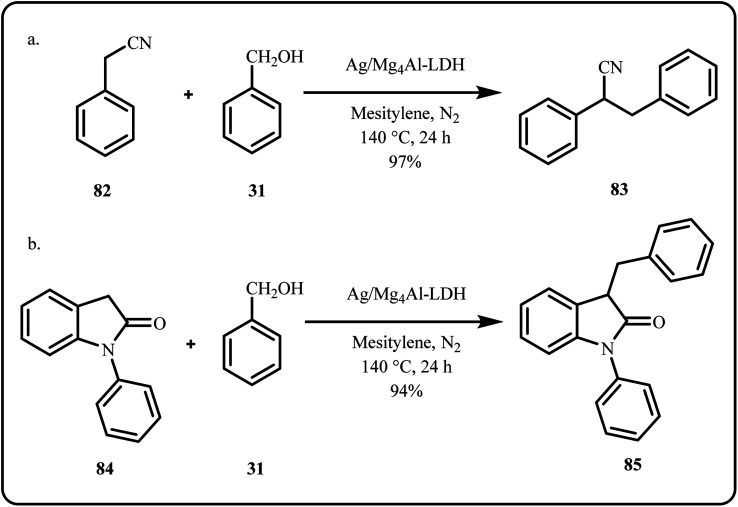
[Ag/Mg_4_Al-LDH] catalyzed α-alkylation of (a) nitriles and (b) oxindoles with alcohols (Aranda *et al.*^41^).

By employing the borrowing hydrogen strategy, a novel heterogeneous silver-catalyst, [Ag/Mg_4_Al-LDH], was utilized for the α-alkylation of nitriles, oxindoles and carboxylic acid derivatives using alcohols (Scheme 25). Additionally, the nanocatalyst facilitated the cyclization of *N*-[2-(hydroxymethyl)phenyl]-2-phenylacetamides to synthesize 3-aryl-quinolin-2(1*H*)-ones *via* one-pot dehydrogenation and intramolecular α-alkylation process.^41^

**Scheme 26:**
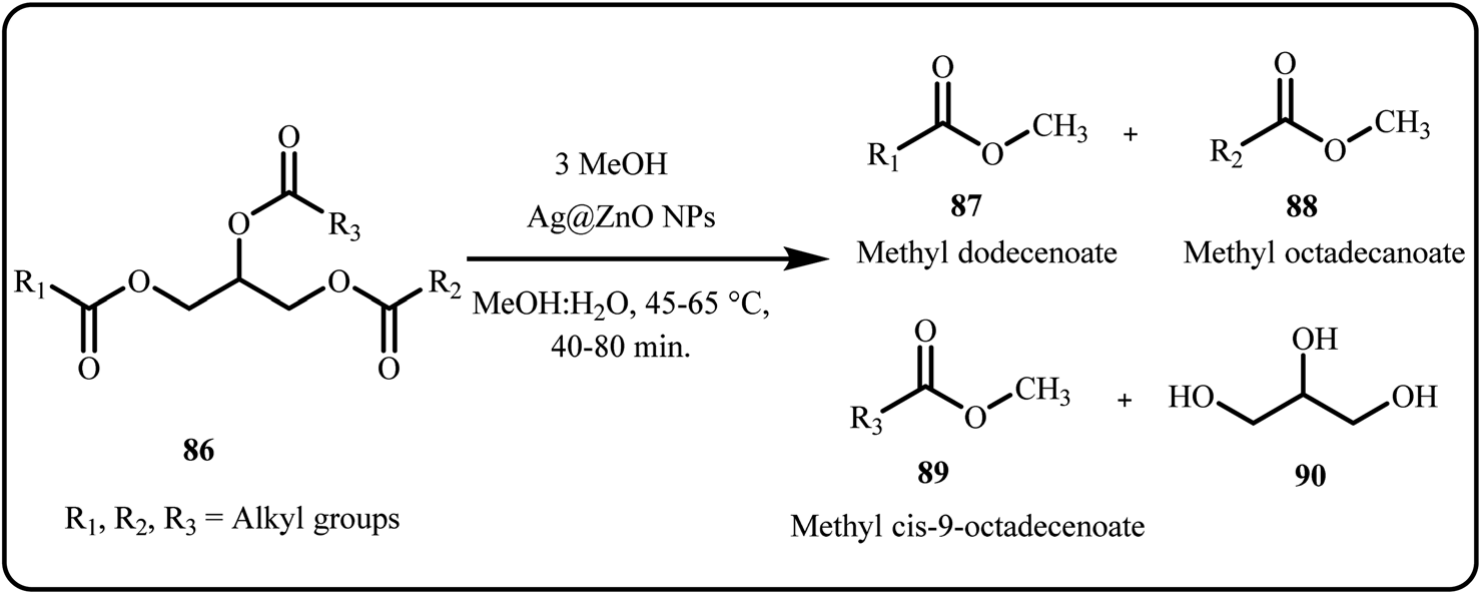
Transesterification of palm oil triglyceride to form biodiesel (FAME) using ZnO@Ag NPs (Laskar *et al.*^164^).

In 2023, Sherif *et al.* developed a green CaO-based eggshell-Ag heterogeneous nanocatalyst to recycle waste cooking oil and convert it into biodiesel. The group prepared the Ag nanoparticles from fenugreek leaf extract and saturated them over eggshell-derived CaO nanoparticles (CaO/Ag). The CaO/Ag catalytic system afforded a 90% biodiesel yield, surpassing the CaO catalytic system attributed to its larger surface area and pore volume.^162^ Recently, Zaban *et al.* produced biodiesel from *Aspergillus* terreus KC462061 in presence of gold–silver nanocatalyst (Au@Ag NPs) achieving a maximum yield of 43% in a fast and safe way.^163^ Biodiesel synthesis through transesterification of palm oil was efficiently catalyzed by Laskar *et al.* by employing a novel ZnO supported Ag nanocomposite (ZnO@Ag NPs) (Scheme 26). Interestingly, the synthesized ZnO@Ag NPs outperformed other catalysts, such as ZnO, Ag, ZnO mixtures, Ag@Al_2_O_3_, and Ag@SiO_2_, achieving an impressive yield of 96%.^164^

**Scheme 27:**
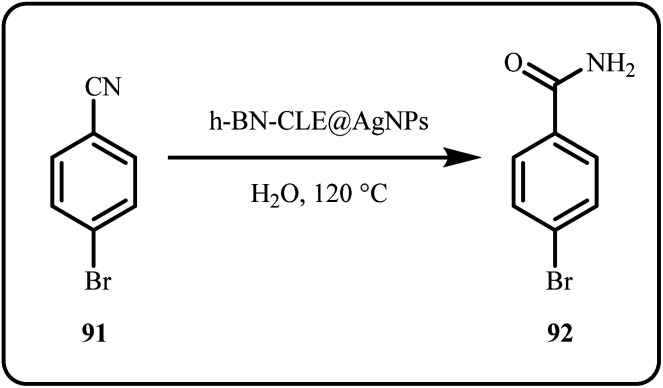
Amidation of nitriles using h-BN-CLE@AgNPs (Antony *et al.*^165^).

Biogenically fabricated Ag NPs, supported on boron nitride (h-BN-CLE@AgNPs) efficiently catalyzed the amidation of nitriles to produce excellent yields of aryl amides (Scheme 27). Furthermore, the nanocatalyst followed an economical and easy synthesis method, and produced non-toxic environment friendly by-products.^165^

**Scheme 28:**
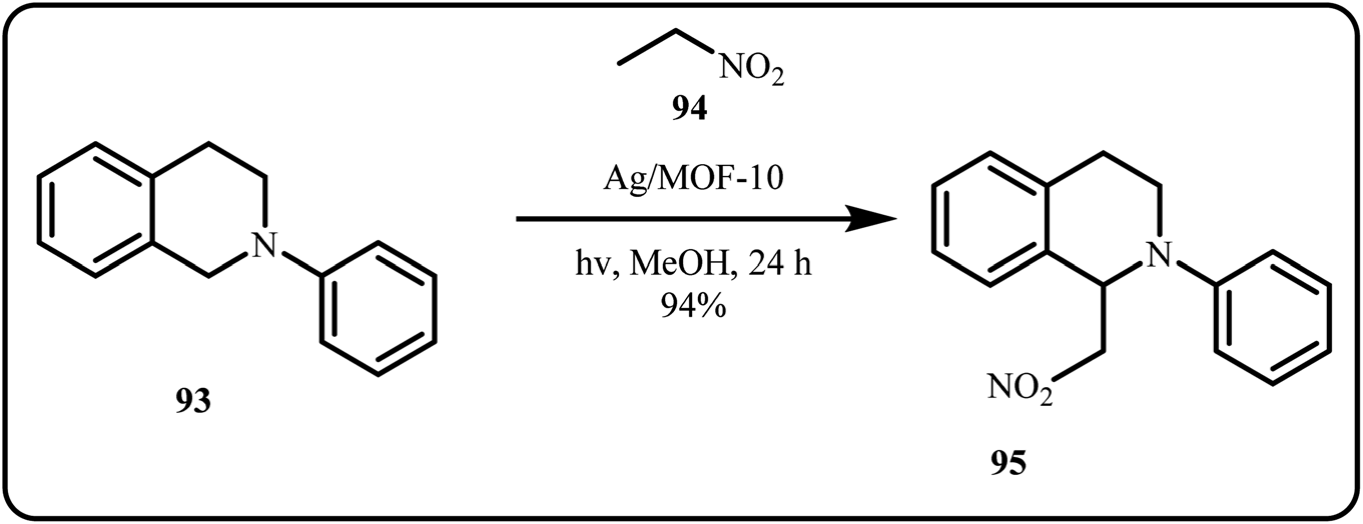
Cross-dehydrogenation coupling reaction photocatalyzed by Ag/MOF-10 (Che *et al.*^44^).

In a recent study, Che *et al.*, developed a novel photocatalyst, Ag/MOF nanocomposite, for cross-dehydrogenation coupling (CDC) reactions (Scheme 28). Using a simple photoreduction method, the Ag nanoparticles were stabilized onto the Metal Organic Framework (MOF) surface, resulting in an efficient and recyclable photocatalyst. The group highlighted that catalytic performance was dependent on nanoparticle size and loading amount, with smaller nanoparticles exhibiting superior activity.^44^

**Scheme 29:**
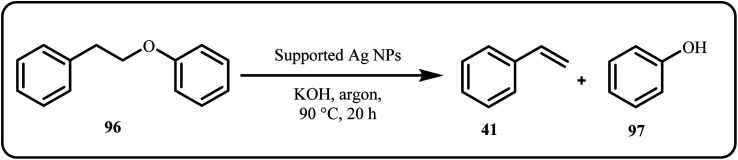
Hydrogen abstraction of benzylic Cα atom for alkyl aryl ether bond cleavage (Han *et al.*^166^).

Recently, Han *et al.* presented a novel strategy that utilized illuminated plasmonic silver nanoparticles (Ag NPs) to efficiently abstract hydrogen from the C(sp^3^)–H bond of the Cα atom in an alkyl-aryl ether β-O-4 linkage under mild conditions (Scheme 29). The proposed plasmon-driven process selectively abstracted hydrogen from specific C(sp^3^)–H bonds and precisely cleaved C–O bonds to produce aromatic compounds with unsaturated, substituted groups in excellent yields.^166^

**Scheme 30:**
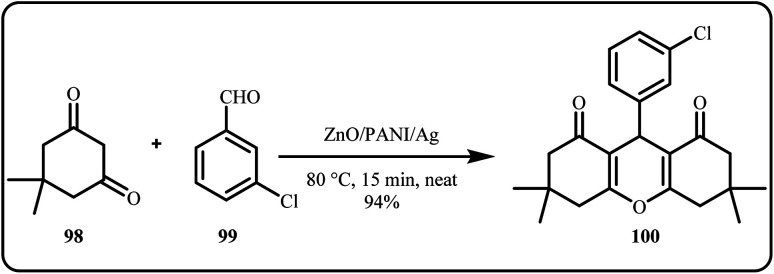
ZnO/PANI/Ag nanocatalyzed synthesis of xanthene-1,8(2*H*)-dione derivatives (Nisha *et al.*^43^).

In this work, Nisha *et al.* developed a novel and highly efficient heterogeneous ZnO/polyaniline (PANI)/Ag nanocomposite,for the catalytic one-pot synthesis of xanthene derivatives *via* the condensation of 5,5-dimethylcyclohexane-1,3-dione with various aromatic aldehydes under solvent-free conditions at 80 °C, achieving complete conversion in just 15 minutes (Scheme 30). The catalyst offered lesser reaction time, easy work-up and excellent catalyst recyclability for up to 6 cycles, while maintaining high yields.^43^

Recently, Karmakar *et al.* developed an efficient protocol for the cross dehydrogenative coupling (CDC) of xanthene and aromatic compounds using a nanosized bimetallic Ni/Ag@titania catalyst. The catalytic activity was studied through the reaction between caffeine and vanillin, comparing four polymorphic forms of titania-brookite, rutile, anatase and mesoporous out of which Ni/Ag@anatase demonstrated the highest catalytic activity, achieving 88% product formation in dry DMSO, attributed to the high surface area of anatase form. The group also revealed that the reaction's regioselectivity was influenced by the selection of peroxide oxidant, with both TBHP and H_2_O_2_ proving effective.^167^

**Scheme 31:**
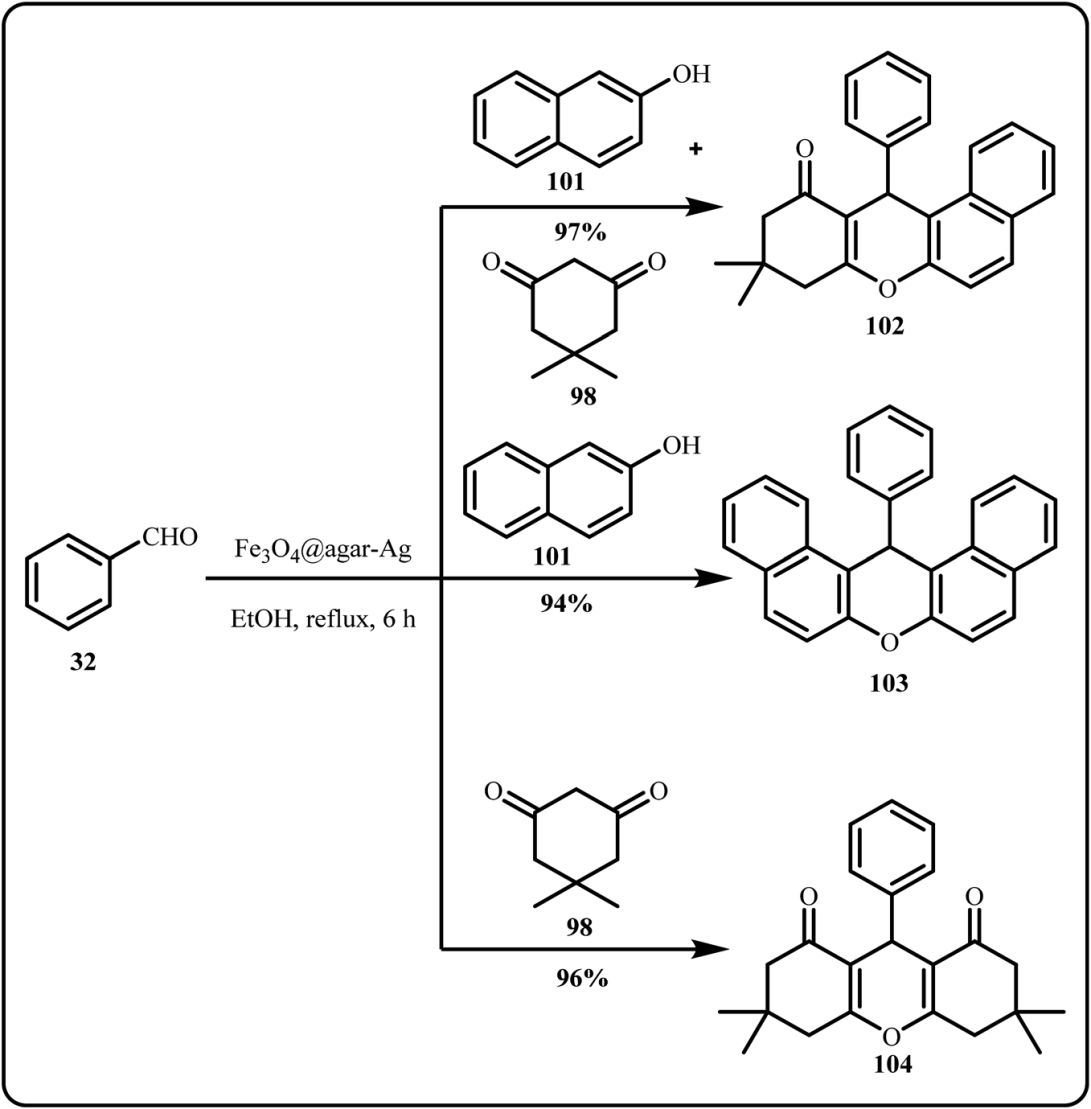
Synthesis of xanthene derivatives using Fe_3_O_4_@Agar-Ag nanocatalyst (Hoseinzade *et al.*^168^).

Hoseinzade *et al.* highlighted the production of a novel magnetically recyclable nanocatalyst, Fe_3_O_4_@Agar-Ag, *via in situ* co-precipitation of Fe^2+^ and Fe^3+^ ions using NH_4_OH in an agar solution, followed by the Ag^+^ ion coating and the subsequent reduction with NaBH_4_. The Fe_3_O_4_@Agar-Ag nanocatalyst demonstrated significant catalytic activity in synthesizing various xanthene derivatives, specifically 12-aryl-8,9,10,12-tetrahydrobenzo[*a*]xanthene-11-one, 14-aryl-14H-dibenzo[*a*,*j*]xanthene and 1,8-dioxo-octahydroxanthene, *via* one-pot condensation of dimedone, aryl aldehydes and 2-naphthol in ethanol (Scheme 31). This method offered simple product isolation, easy catalyst recovery and high yields under eco-friendly conditions, while also eliminating the use of toxic catalysts.^168^

**Scheme 32:**
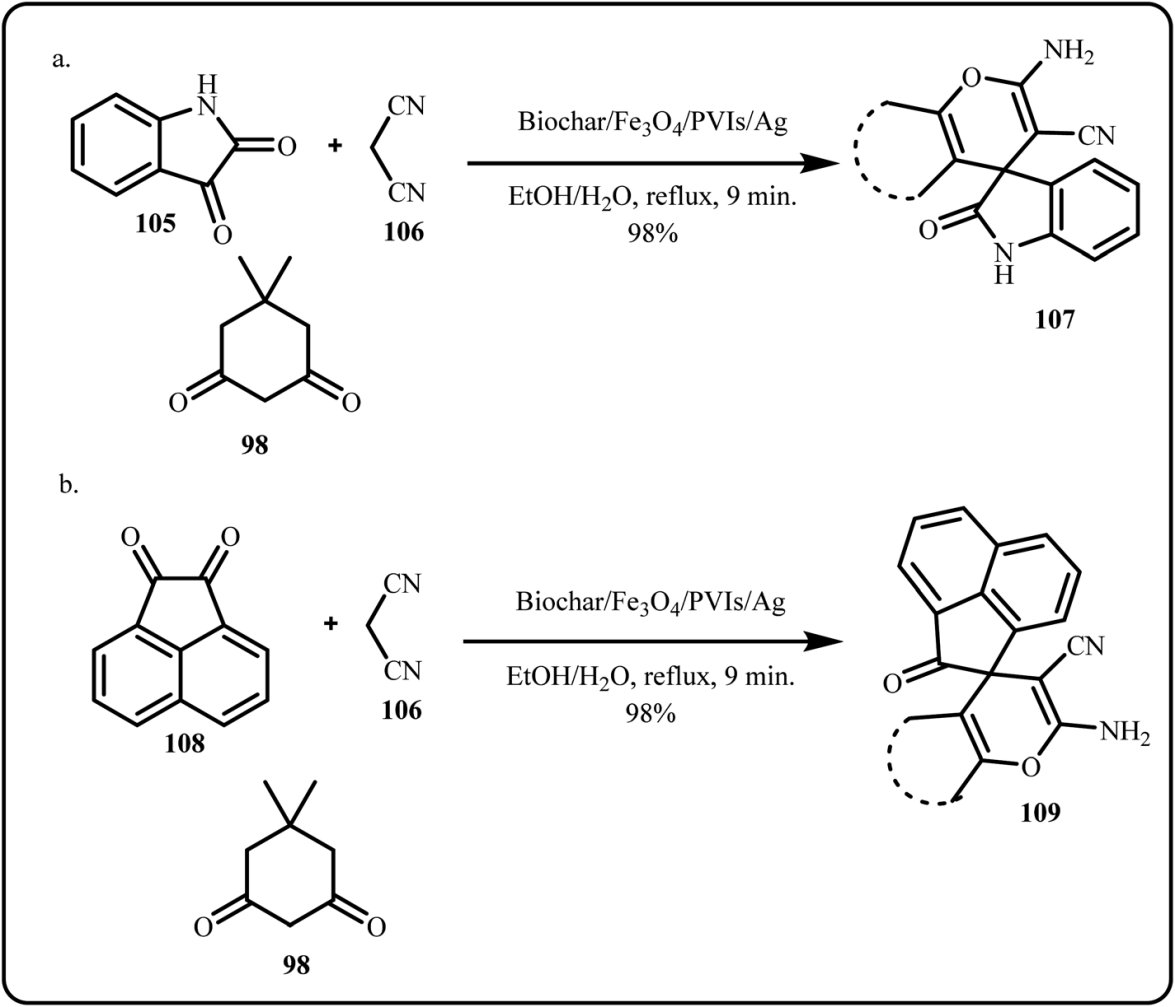
Biochar/Fe_3_O_4_/PVIs/Ag catalyzed synthesis of spiro-2-amino-4*H*-pyran compounds using (a) isatin and (b) acenaphthenequinone (Mohammadi *et al.*^169^).

A novel silver nanoparticle catalyst was synthesized by developing poly(1-vinylimidazole) on the surface of magnetic biochar derived from Spear Thistle (biochar/Fe_3_O_4_/PVIm/Ag NPs). The group investigated the catalytic efficiency of the generated nanocatalyst through multicomponent reactions, focused on synthesizing spiro-2-amino-4*H*-pyrans *i.e.* spirochromenes (Scheme 32). The nanocatalyst offered high stability and reusability while affording high product yields in mild eco-friendly reaction conditions.^169^

**Scheme 33:**
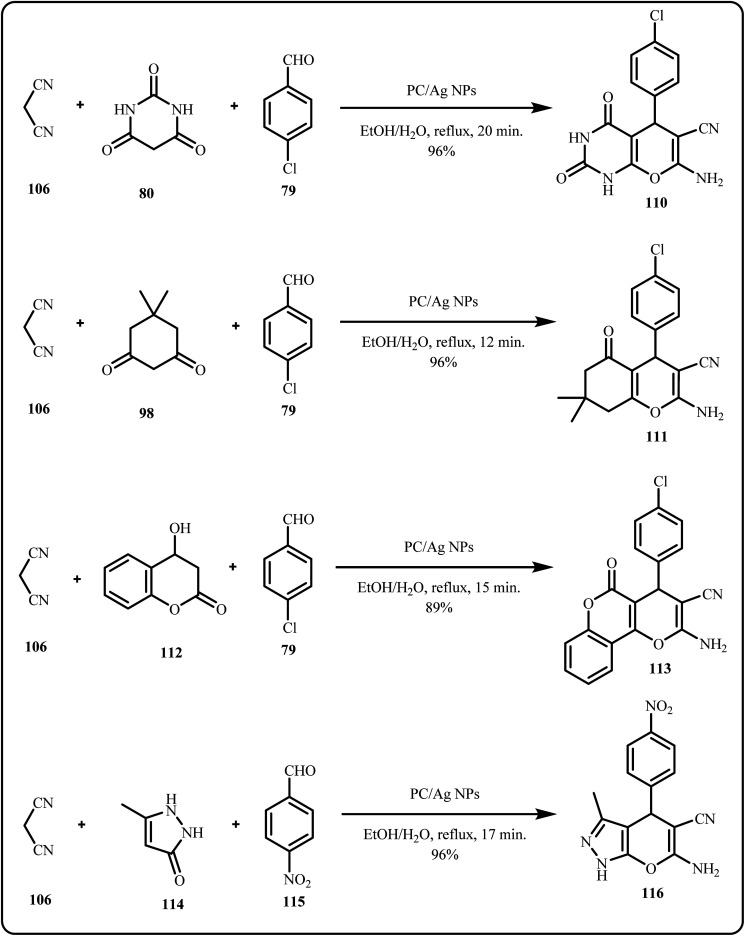
Catalytic synthesis of 2-amino-4*H*-pyran and functionalized spirochromene derivatives using PC/Ag NPs (Saneinezhad *et al.*^170^).

Recently, a novel reuseable green silver based biocomposite (PC/AgNPs) was prepared and tested for the catalytic synthesis of 2-amino-4*H*-pyran and functionalized spirochromene derivatives *via* a one-pot, multicomponent reaction (Scheme 33). The prepared catalyst demonstrated high efficiency, easy separation, and reusability for at least three cycles without significant loss of activity.^170^

**Scheme 34:**
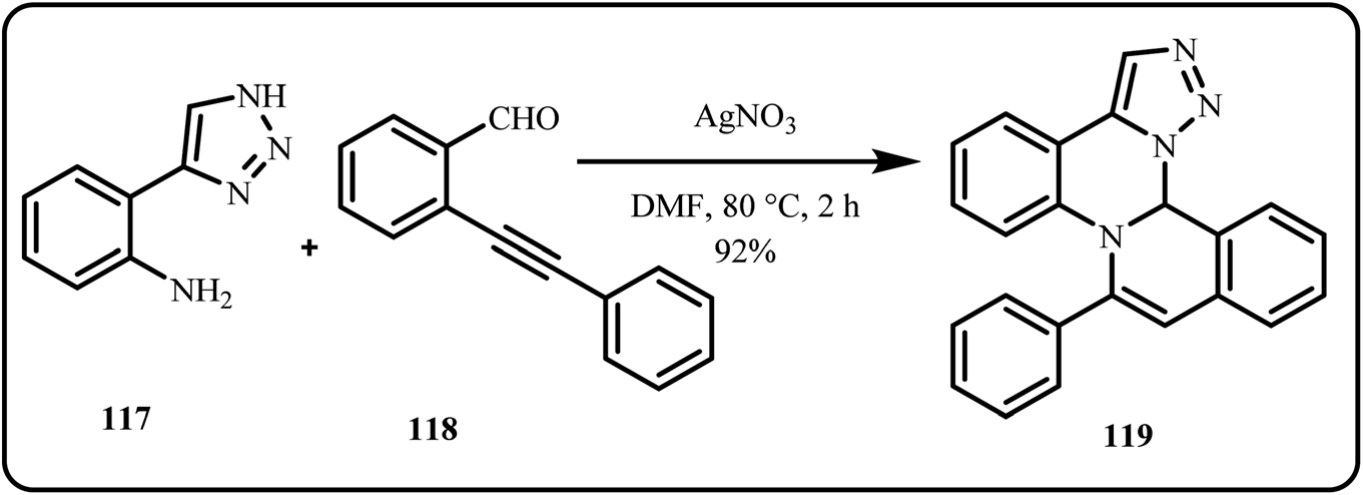
AgNO_3_ catalyzed cyclization of amino-NH-1,2,3-triazoles with 2-alkylbenzaldehyde to give pentacyclic fused triazoles (Zhang *et al.*^171^).

Recently, Zhang *et al.* developed an Ag(i)-catalyzed synthetic route to produce novel isoquinoline and quinazoline fused 1,2,3-triazoles in good-to-excellent yields (Scheme 34). Mechanistically, the reaction proceeds through condensation and amination cyclization cascade of amino-NH-1,2,3-triazoles with 2-alkynylbenzaldehydes forming three new C–N bonds in a single step, where the –NH group of the triazole ring acts as a nucleophile to give the quinazoline skeleton.^171^

**Scheme 35:**
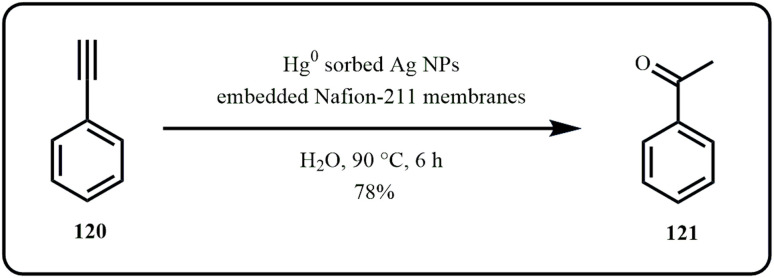
Catalytic transformation of phenylacetylene to acetophenone using Hg^0^ embedded nafion-211 membrane (Yakkala *et al.*^172^).

Yakkala *et al.* developed Ag NPs embedded in a poly(perfluorosulfonic) acid cation-exchange membrane (Nafion-211), followed by the sorption and preconcentration of Hg^2+^ ions *via* a galvanic reaction that resulted in Hg^0^ nanodroplets. Interestingly, the Hg^0^ embedded membrane served as a dip catalyst for converting phenylacetylene to acetophenone (Scheme 35). Additionally, the membrane successfully quantified Hg^2+^ in real water samples and exhibited potential for safe Hg storage for remediation purposes.^172^

**Scheme 36:**
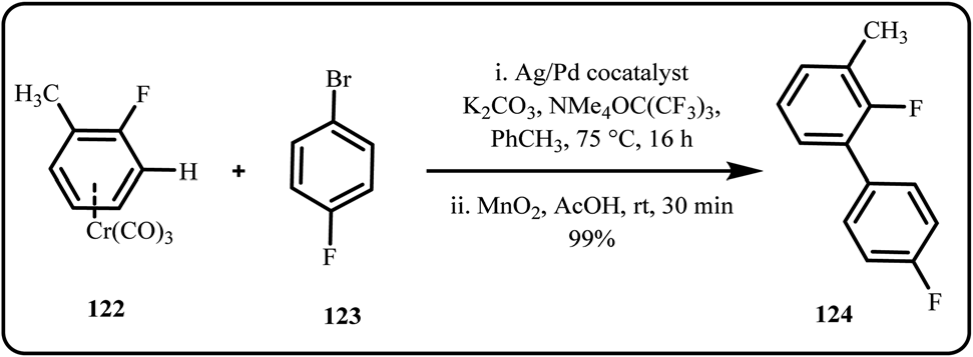
Ag/Pd co-catalyzed arylation of fluoroarene derivatives with aryl bromides (Panigrahi *et al.*^173^).

A novel Ag/Pd cocatalyst was employed for direct C–H arylation of fluoroarene chromium tricarbonyl complexes with bromoarenes (Scheme 36). Herein, the catalytic system operated under mild conditions, where Ag(i) facilitated C–H activation, while Pd promoted oxidative addition and reductive elimination in bromoarene,enabling the successful arylation of regioselective fluoroarenes, further enhanced by π-complexation to Cr(CO)_3_.^173^

**Scheme 37:**
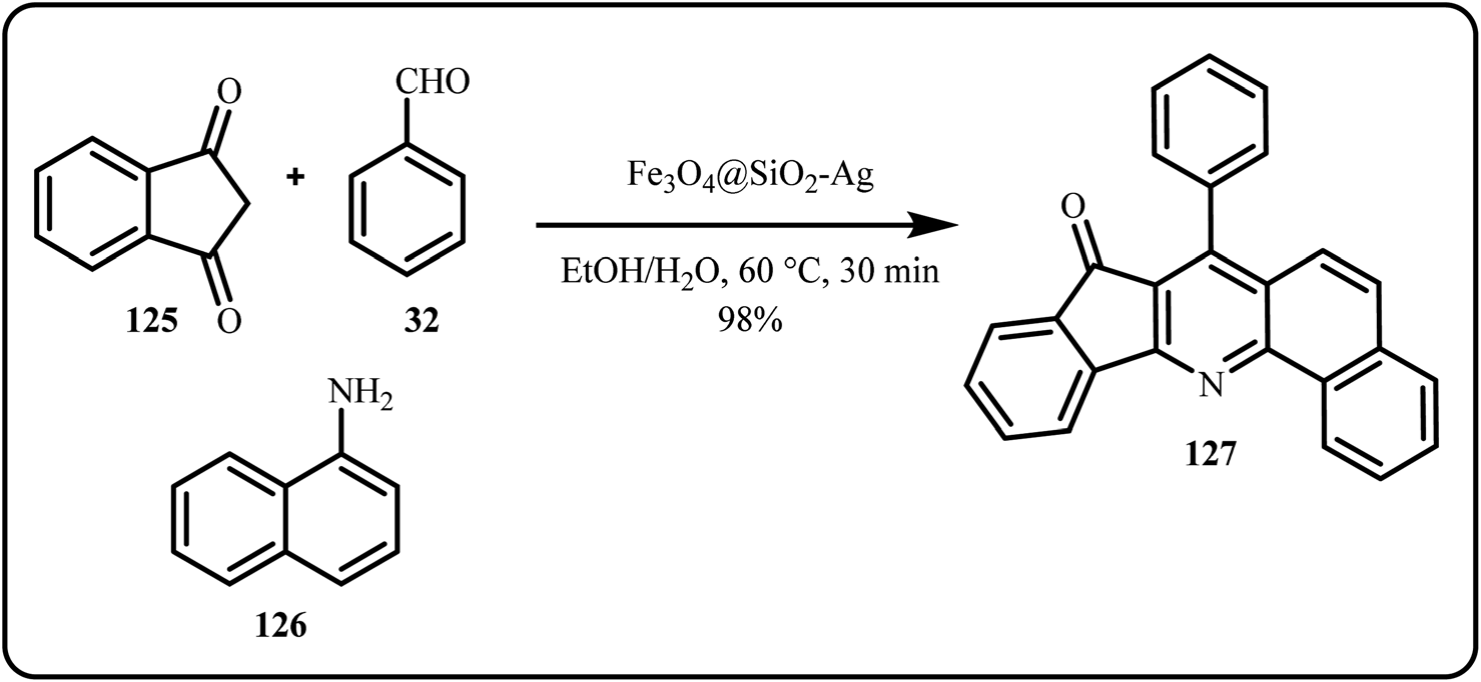
Catalytic synthesis of quinoline heterocyclic derivatives using Fe_3_O_4_@SiO_2_-Ag (Singh *et al.*^174^).

An interesting study by Singh *et al.*, highlighted the use of Fe_3_O_4_@SiO_2_-Ag nanocatalyst for the synthesis of quinoline heterocyclic derivatives (Scheme 37). Herein, the group coupled aldehyde, amine and 1,3-indanedione to get high yields of products in a shorter time frame.^174^

**Scheme 38:**
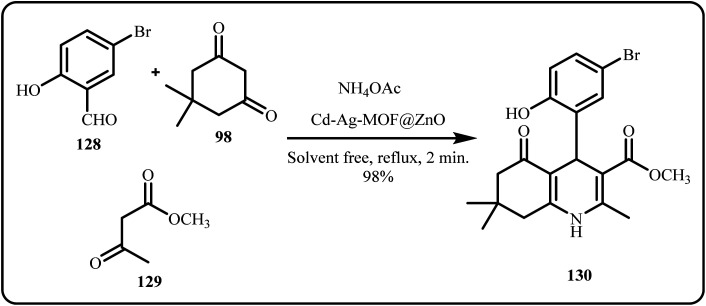
Catalytic synthesis of polyhydroquinoline heterocycle derivatives using Cd-Ag-MOF@ZnO (Hoot *et al.*^175^).

Similarly, Hoot *et al.* prepared polyhydroquinoline heterocycles with Cd-Ag-MOF@ZnO nanoribbon as the organocatalyst *via* a one-pot Hantzsch condensation under solvent free conditions (Scheme 38). Herein, the products were obtained through a highly efficient and time-saving method in high yield.^175^

**Scheme 39:**
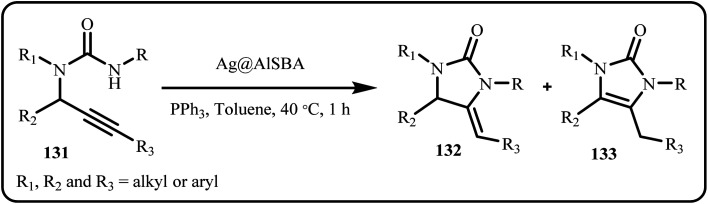
Synthesis of imidazolones *via* cycloisomerizations of propargylic ureas (Zuliani *et al.*^177^).

Co *et al.* developed binary nickel–silver nanoparticles, supported them on zeolite NaA and investigated the synthesized catalytic system for the dehydrochlorination of 2,4-dichlorophenol and observed a high conversion rate of 91% by using 10%NiAg/ZA.^176^ Zuliani *et al.* prepared imidazolones *via* the cyclo isomerization of propargylic ureas through a novel, environmentally friendly approach that employed heterogeneous catalysis with Au and Ag NPs supported on AlSBA-15 (Scheme 39). Additionally, the group conducted and compared the reactions with conventional and microwave irradiation reaction conditions, wherein microwave heating significantly reduced the reaction time.^177^

In their recent study, Hammouda *et al.* utilized a biosynthesized Ag–TiO_2_ nanocatalyst by using turmeric ethanol extract as the reducing and chelating agent to synthesize novel benzopyrimido[4,5-*d*]azoninone derivatives, achieving yields ranging from good to excellent (57–91%) and also showed good antioxidant activity.^178^

**Scheme 40:**
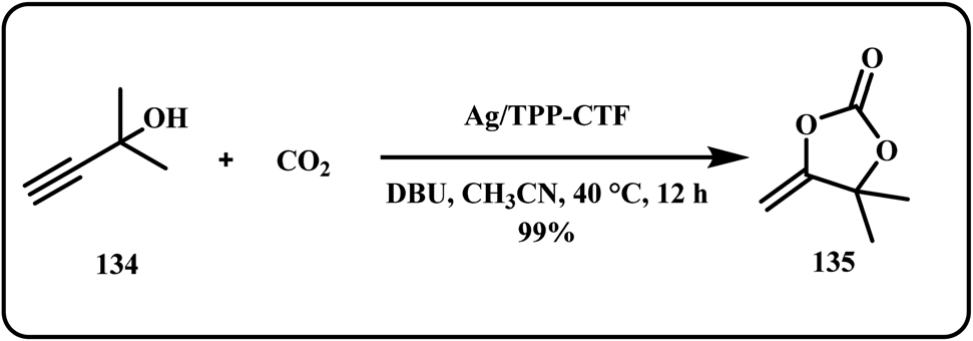
Synthesis of α-alkylidene cyclic carbonates *via* CO_2_ insertion using propargyl alcohols (Yang *et al.*^182^).

Over years, many researchers have opted CO_2_ capture and insertion in order to synthesize valuable organic compounds through various catalysts.^179–181^ For example, Yang *et al.* utilized Ag NPs anchored onto triazine-based framework for catalytic CO_2_ conversion into α-alkylidene cyclic carbonates using propargyl alcohols at room temperature (Scheme 40). Interestingly, the N-rich dual active sites worked as both the electron acceptor and donor. Herein, the synthesized catalyst can be easily recovered and reused for at least 10 consecutive cycles while offered high catalytic activity of up to 99%.^182^

**Scheme 41:**
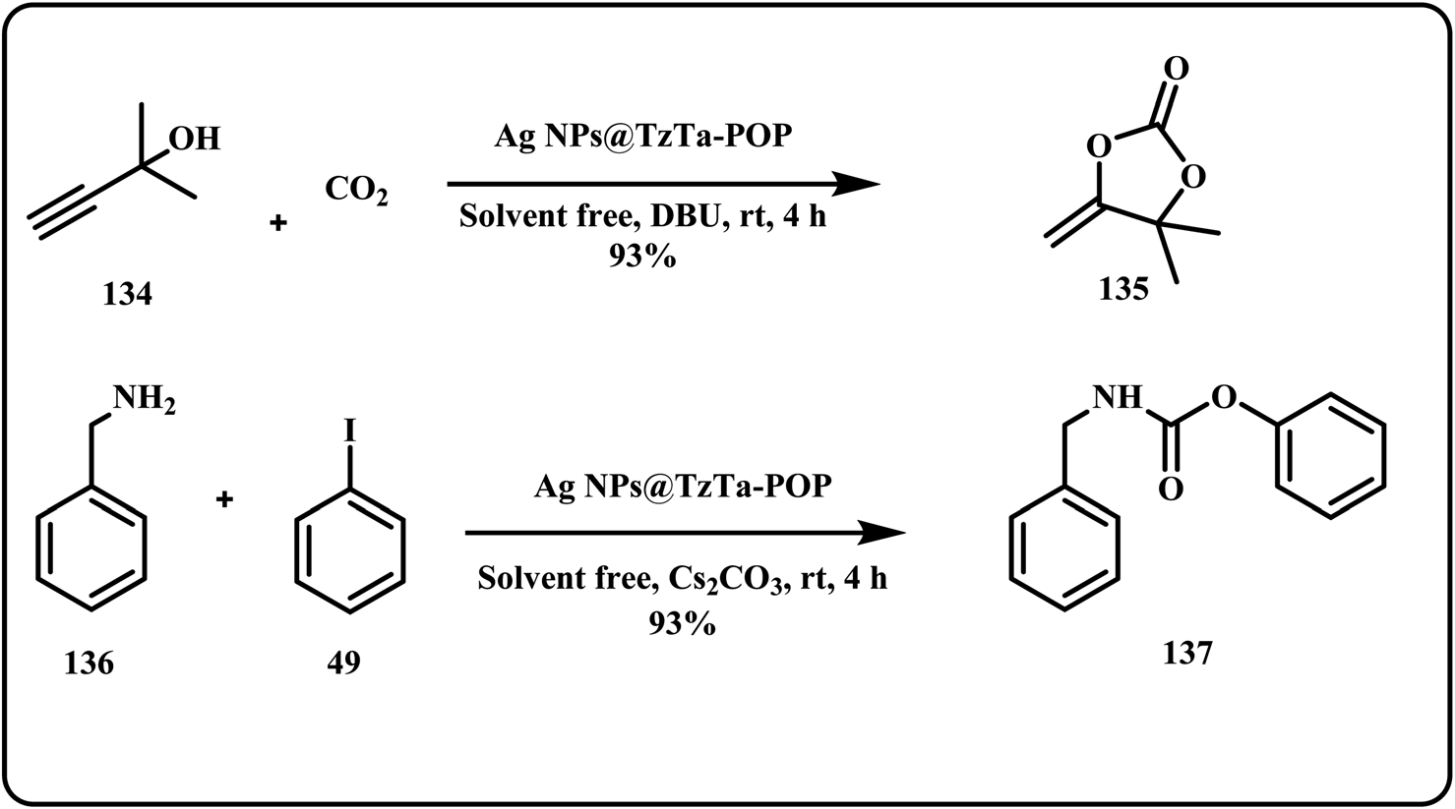
Ag NPs@TzTa-POP catalyzed synthesis of α-alkylidene cyclic carbonates and value added carbamates (Roy *et al.*^183^).

Similarly, Roy *et al.* catalyzed carboxylative cyclization of propargyl alcohols to produce α-alkylidene cyclic carbonates at room temperature with significant yield of 87–98% and >99% selectivity (Scheme 41). Furthermore, the group synthesized high yield (87–95%) of value-added carbamates from aromatic and aliphatic amines, halides and CO_2_.^183^

**Scheme 42:**
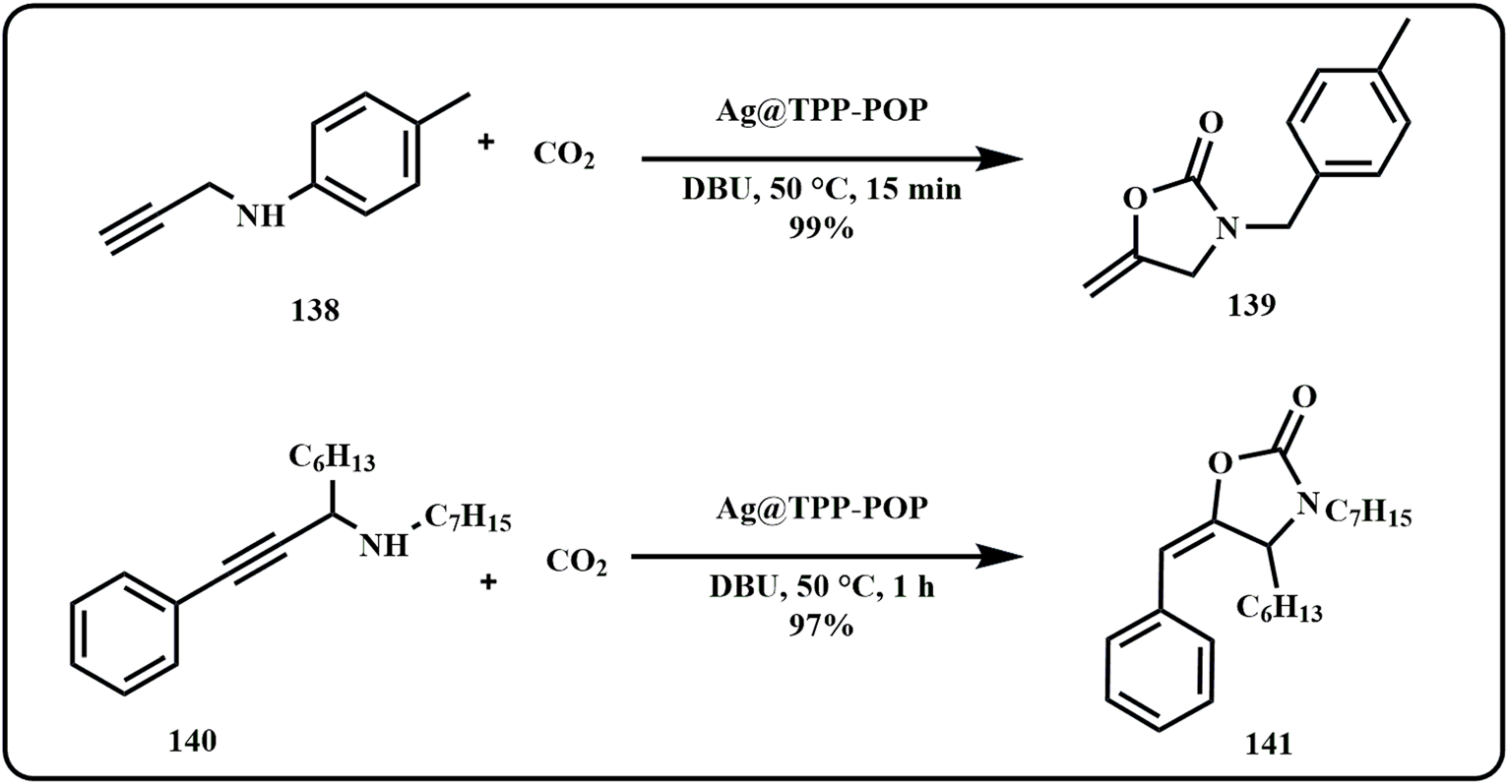
Synthesis of oxazolidinone derivative from propargylic amines using Ag NPs@TzTa-POP catalytic system (Sahoo *et al.*^184^).

On a similar note, recently Sahoo *et al.* developed an Ag-based catalytic system that efficiently converted terminal and internal propargylic amines into their respective oxazolidinone derivatives, offering high yield of products (Scheme 42).^184^

**Scheme 43:**
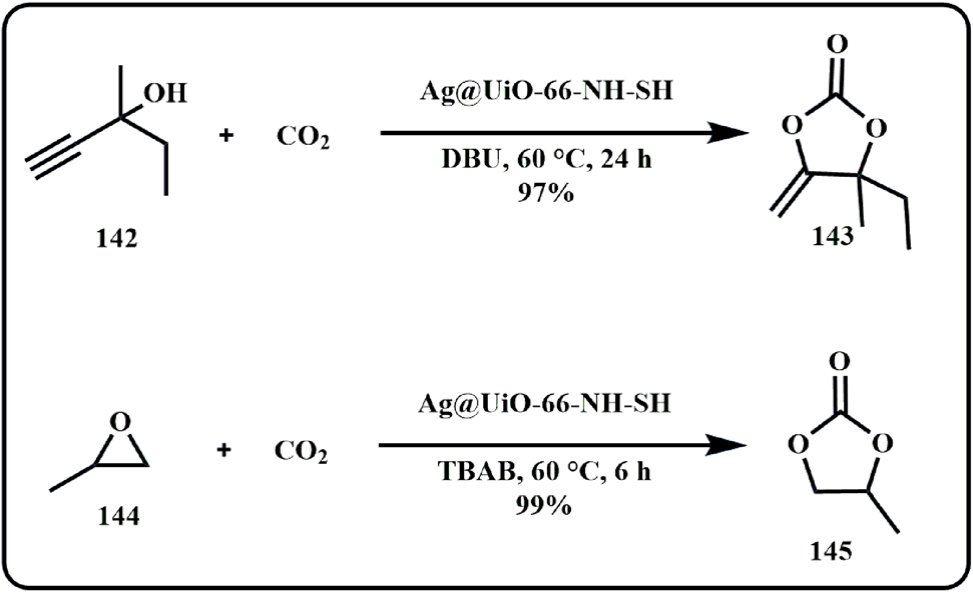
Synthesis of cyclic carbonates using propargylic alcohols and epoxides using Ag@UiO-66-NH-SH catalytic system (Patra *et al.*^185^).

Recently, Patra *et al.* designed an Ag NP modified thiol MOF-based catalytic system that converted propargylic alcohols and terminal epoxide to respective cyclic carbonates (Scheme 43). Herein, the group exploited the soft–soft interaction between free standing thiol group and Ag to make multiple catalysts by varying Ag concentration that offered high yield products for both the reactions.^185^

**Scheme 44:**
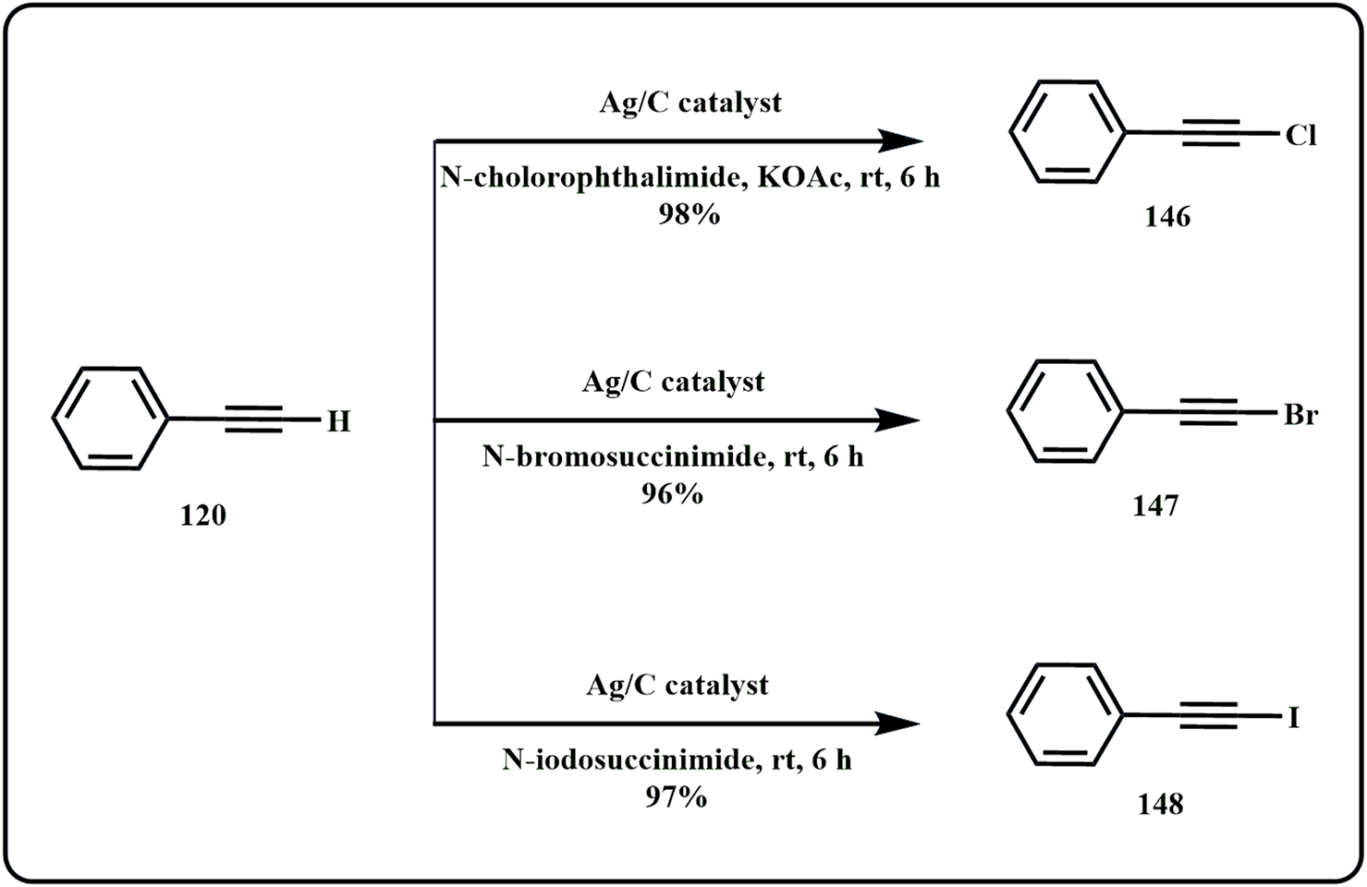
Alkyne halogenation catalyzed by Ag/C catalytic system (Liu *et al.*^186^).

In their recent study, Liu *et al.*, designed a silver/carbon nanocatalyst utilising *Rhizoma coptidis* root as a support for the terminal alkyne halogenation reaction (Scheme 44). Interestingly, the catalyst achieved a catalytic yield of approximately 90% with high stability and reusability for up to 5 cycles.^186^

**Scheme 45:**
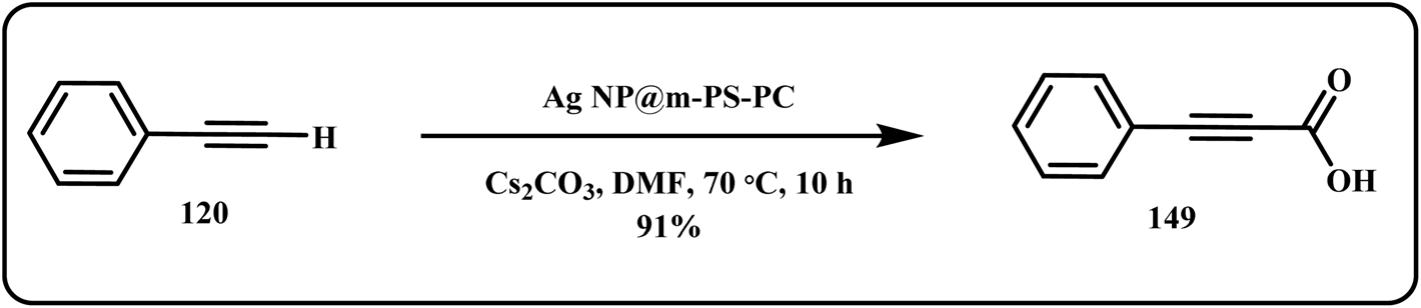
Carboxylation of monosubstituted alkynes using Ag NP@m-PS-PC catalyst (Salam *et al.*^40^).

Similarly, Salam *et al.* reported a silver-based catalytic system (Ag NP@m-PS-PC) for the carboxylation of monosubstituted alkynes to give high yields of respective carboxylic acids without any decline in the catalytic activity for up to 5 cycles (Scheme 45).^40^

**Scheme 46:**
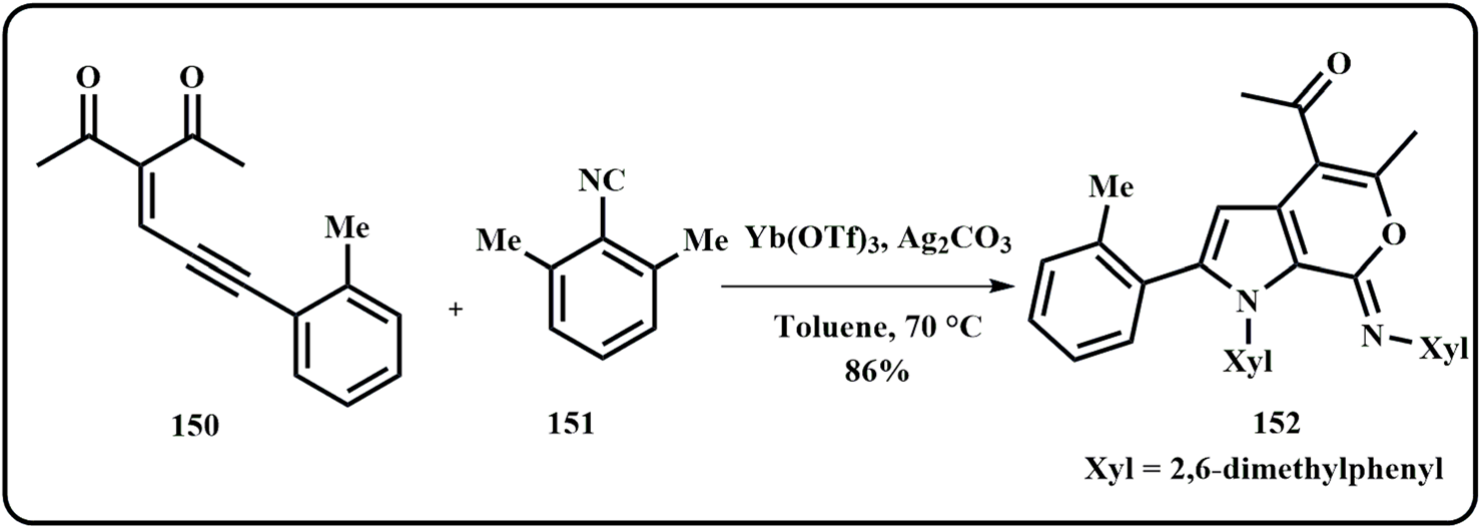
Synthesis of pyrrole-fused heterocycles using ytterbium and silver co-catalyst (Li *et al.*^187^).

Li *et al.* employed ytterbium and silver co-catalyst to prepare pyrrole-fused heterocycles using isocyanide and enynone (Scheme 46). Interestingly, the versatile catalyst sustained various functional groups in the reaction and offered great yields of products.^187^

**Scheme 47:**
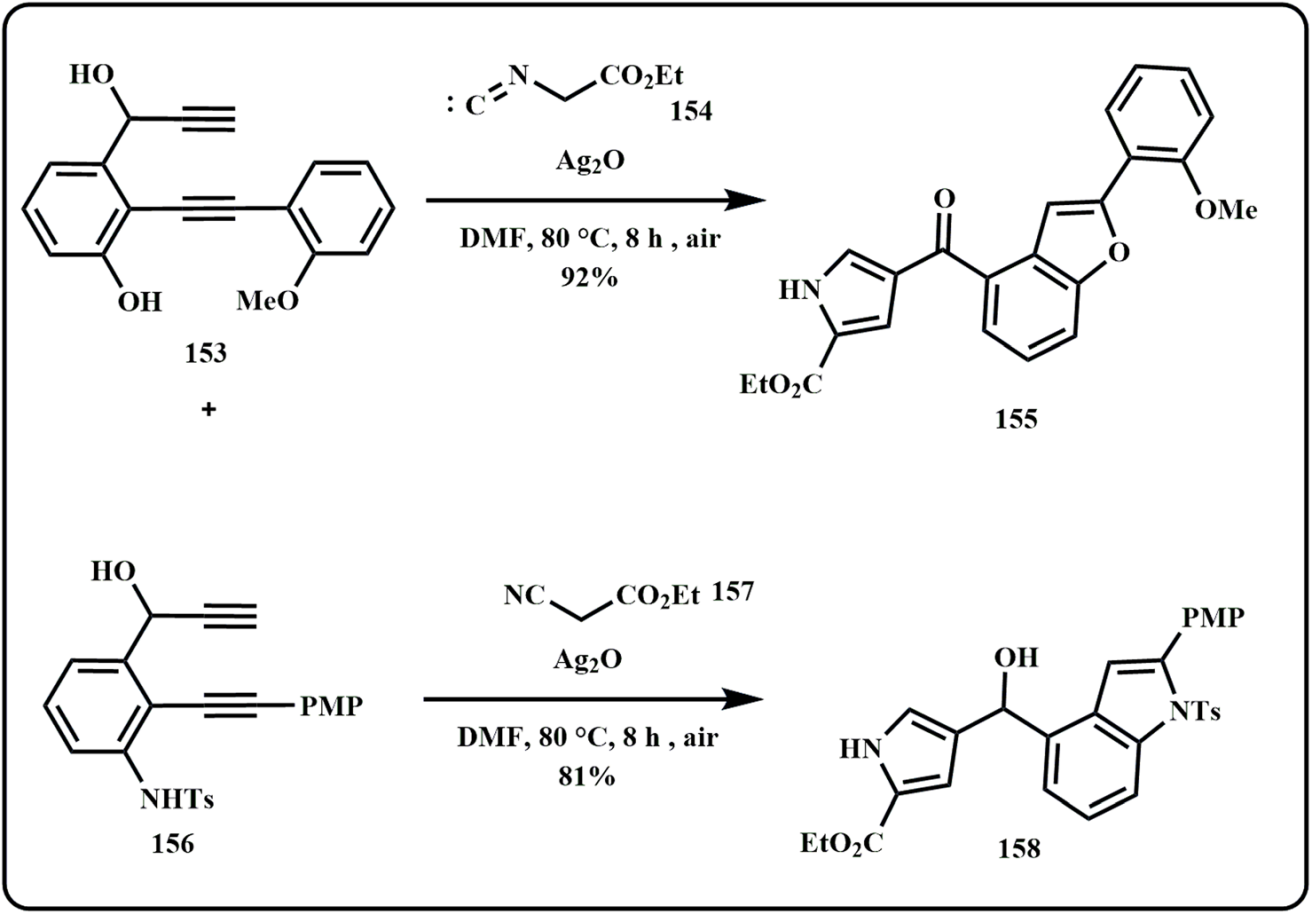
Synthesis of benzofuran-pyrroles and indole–pyrroles using Ag_2_O catalyst (Liu *et al.*^188^).

An interesting study by Liu *et al.* highlights the use of silver based nanocatalyst to prepare various substituted benzofuran-pyrroles. Not only this, the group also catalytically synthesized indole–pyrrole using the same catalyst (Scheme 47).^188^

**Scheme 48:**
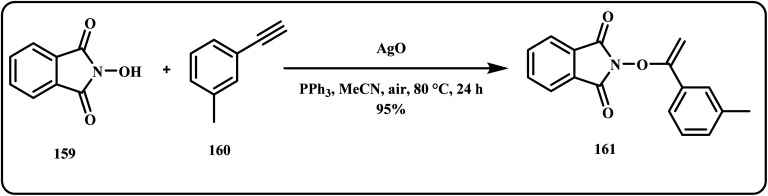
Synthesis of *N*-enoxyimides by hydrooxyimidation of terminal alkynes using AgO catalyst (Noor *et al.*^189^).

Recently, Noor *et al.* demonstrated the AgO catalyzed synthesis of *N*-enoxyimides *via* hydrooxyimidation of terminal alkynes (Scheme 48). Herein, the catalyst offered exceptional yields of 96% under mild reaction conditions *via* a simple, scalable and atom efficient method.^189^

## 3. Biomedical applications

**Fig. 6:**
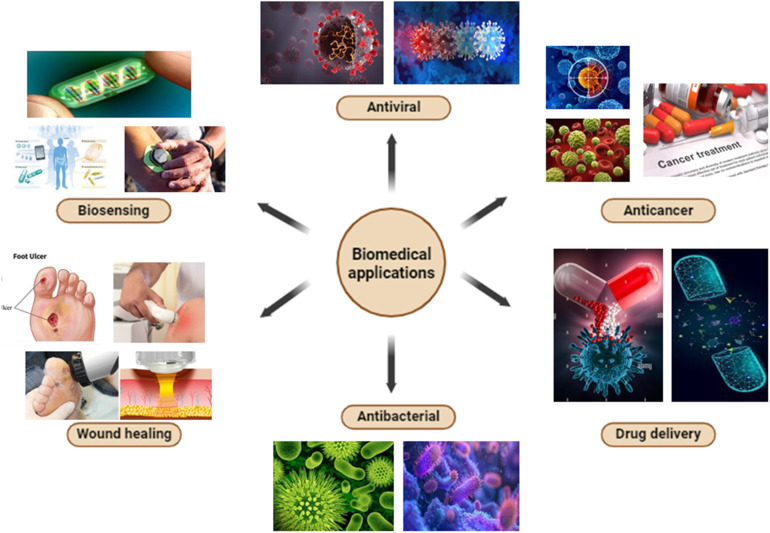
Biomedical applications of silver nanocomposites.

Earlier studies in the literature have shown that Ag NPs possess remarkable antiviral, antifungal, antibacterial, antifouling, and antioxidant properties, which enhance the biocompatibility of silver-based nanomaterials. Attributed from these interesting features, many such nanomaterials are heavily employed for the advancements in biomedical fields^190^ such as drug delivery, wound healing and biosensing (Fig. 6).

### 3.1 Antibacterial

Ag-based nanocomposites have attracted considerable attention for their strong antibacterial properties, making them highly valuable in both biomedical and industrial fields. The unique characteristics of Ag NPs, such as their high surface area^191^ and ability to release Ag^+^ ions,^192^ enable effective interaction with bacterial cells.^193^ A typical antibacterial mechanism involves the production of reactive oxygen species (ROS),^194^ which cause oxidative stress and damage to the bacterial membranes,^195^ proteins, and DNA.^196^ Verma *et al.* developed ZnO–Ag nanocomposites by employing solvothermal method and evaluated its antibacterial capabilities through agar well diffusion assay. The antibacterial efficacy of the nanocomposite was observed to have improved with respect to individual nanoparticles, particularly against *E. coli*. When exposed to light, ZnO released toxic substances that killed the germs, while silver enhanced the antibacterial properties.^197^ Slewa *et al.* developed AgNPs@CQD, a low toxicity nanomaterial synthesized using ecofriendly onion juice. Tested *via* the agar-well diffusion method, it showed strong antibacterial activity, especially at higher concentrations of CQDs.^198^ Rabbi *et al.* synthesized Fe_2_O_3_/Ag, an antibacterial nanocomposite, and investigated its effectiveness against four pathogenic bacterial species.^199^

Similarly, Muneeswaran *et al.* developed CS/Ag nanocomposites *via* starch-mediation. Both Ag NPs and CS/Ag NCs exhibited antibacterial efficacy against *Salmonella typhi*, *E. coli*, *Pseudomonas aeruginosa*, and multidrug-resistant *Klebsiella pneumoniea*, with minimum inhibitory concentrations ranging from 1.3 to 7.8 μg mL^−1^.^200^ Recently, Song *et al.* formulated silver nanoparticles supported on attapulgite clay to develop a new antibacterial material, which successfully eradicated bacteria like *E. coli*.^201^ Bharathi *et al.* utilized gum arabic (GA) and Chitosan (CS) to manufacture sustainable and environmentally friendly silver nanocomposites that demonstrated promising inhibition against *Staphylococcus aureus* (18 mm) and *Escherichia coli* (20 mm).^202^ Arunpandian *et al.* used a facile hydrothermal route to fabricate Ag/Er_2_O_3_@CuO, a novel nanocomposite that successfully destroyed hazardous bacteria such as *Aeromonas hydrophila* and *Hemophilus influenza*.^203^

### 3.2 Anticancer

According to multiple reports, U.S. is expected to have over 2 million cancer cases and more than 611 000 related deaths in 2024. Since 1991, rising incidence rates of cancers like breast, prostate, and liver have posed a threat to ongoing progress. Additionally, colorectal and cervical cancers are on the rise among younger adults, with colorectal cancer topping the cancer death charts for men under 50.^204^ Ag-based nanocomposites are becoming more and more popular due to their possible uses in the treatment of cancer. Resulting from its nano-size and unique surface properties, they penetrate into the tumor tissues, and effectively target the cancer cells.^205^ Ag NP generate ROS, which induce oxidative stress and ultimately lead to the death of cancer cells. Additionally, they can be combined with other materials, such as medicines, polymers, or graphene, to increase their therapeutic benefits.^206^ This makes them a promising approach for cancer treatment, offering fewer side effects and improved selectivity. Recently, Faid *et al.* synthesized GO/Ag NC, which was tested against four different types of cancer cells, H460, HCT116, MDA-MB-468, and FaDu to assess its capacity to kill cancer cells with IC_50_ values of 5.5, 6, 9, 7.75 μg mL^−1^, respectively.^207^ Similarly, Vankatraman *et al.* used *Morinda citrifolia* leaf extract to create ZnO/Ag NCs that exhibited greater anticancer activity against A549 lung cancer cells, having an IC_50_ 242 μg mL^−1^, as opposed to 398 μg mL^−1^ for ZnO nanoparticles. Moreover, ZnO/Ag NCs and ZnO NPs were also tested for toxicity against non-cancerous RAW264 macrophage cells, demonstrating IC_50_ values of 402 μg mL^−1^ and 494 μg mL^−1^ respectively.^208^ Moghayedi *et al.* designed and developed silver–graphene oxide nanocomposites (Ag–GO) which showed anticancer properties by targeting glioblastoma (U87MG) cancer cells, having an IC_50_ value of 270 μg mL^−1^.^209^

In a recent study, Zhou *et al.* created a silver-coated magnetic nanoparticles (Fe_3_O_4_/Ag NPs) using pomegranate peel extract, that effectively eliminated ovarian cancer cells NIH: OVCAR-3, ES-2, and TOV-21 G.^210^ D. Bharathi *et al.* manufactured chitosan/silver nanocomposites (Kf-CS/Ag) which demonstrated strong activity against triple-negative breast cancer (TNBC) cells, especially the MDA-MB-231 cell line, possessing IC_50_ value of 53 μg mL^−1^.^211^ Ag NPs coated with reduced graphene oxide (AgNPs@rGO), developed by Balaji *et al.*, exhibited a potent anticancer effect, particularly against MCF-7 breast cancer cells. The green produced Ag NPs and AgNPs@rGO demonstrated significant cytotoxicity, with IC_50_ equal to 100 μg mL^−1^ and 108 μg mL^−1^, respectively.^212^

### 3.3 Antifungal

Antifungal agents are compounds that prevent the growth of or eliminate fungi, helping in the treatment and prevention of fungal infections in humans, animals, and plants. They work by disrupting fungal cell membranes, inhibiting cell wall formation, or interfering with DNA and protein synthesis.^213–215^ Ag nanocomposites, known for their potent antifungal properties, are effective against numerous fungal strains and are being explored for applications in medical treatments, coatings, and agriculture for their potent antifungal properties.

Arumugam *et al.* realized silver-embedded carbon nitrides (Ag@g-CN) that exhibited strong antifungal effects against *Candida albicans*, a common pathogen that causes oral infections and showed MIC values ranging from 16 to 256 μg mL^−1^.^216^ Salem *et al.* developed a carboxymethyl cellulous and silver nanoparticles (CMC-AgNP) composite, which displayed strong activity against filamentous fungus, including *Aspergillus fumigatus*, *A. niger*, and *A. terreus*, making it a potent antifungal drug for pharmaceutical settings.^217^ Selvi *et al.* created reduced graphene oxide (Ag/rGO) nanocomposites that exhibited strong antifungal action, especially against *Candida* species, such as *Candida albicans*, *Candida krusei*, and *Candida tropical*.^218^

Chowdhury *et al.* developed Cu and Ag NP nanocomposites that demonstrated notable antifungal activity against *Rhizoctonia solani*, the causative agent of sheath blight in rice.^219^ Tran *et al.* synthesized lignin@Ag/SiO_2_ NPs that demonstrated significant antifungal activity against *Aspergillus flavus*.^220^ Muzio *et al.* created a thin film nanocomposite incorporating AgNPs synthesized using a green route that exhibited strong antifungal activity against 16 clinical isolates from five different *Candida* species.^221^

### 3.4 Antiviral

Silver nanocomposites demonstrate significant antiviral activity owing to their distinctive physical and chemical properties.^222^ Their composites, often incorporating AgNPs with other substances, can inactivate viruses through mechanisms like oxidative stress generation, disruption of viral replication, and binding to viral proteins.^223,224^ Their uses extend across diverse areas such as medical devices,^225^ surface coatings,^226^ and therapeutic treatments,^227^ positioning them as effective solutions for fighting viral infections and improving health outcomes.

The antiviral characteristics of Ag NPs synthesized using *Nigella arvensis* extract were investigated by Elnosary *et al.* wherein, the Ag NPs demonstrated effectiveness against the HSV-1, HAV, and adenovirus, inhibiting their proliferation by 53.6%, 86%, and 17.3%, respectively.^228^ The antiviral activity of green-synthesized Ag_2_O particles (IC_50_ = 0.618 μg mL^−1^) generated by Asif *et al.* was significantly higher compared to chemically synthesized Ag_2_O particles (IC_50_ = 6.129 μg mL^−1^).^229^ Bhatia *et al.* developed metal oxide-based silver nanocomposites, Ag/NiO (AN) (IC_50_ = 3.277 μg mL^−1^) and Ag_2_O/NiO/ZnO (A/N/Z) (IC_50_ = 2.828 μg mL^−1^), to test their antiviral activity through plaque reduction assays, cytopathic effect analysis, and qRT-PCR showing a significant reduction in chikungunya virus titer.^230^

Chitosan NPs (CS NPs) and Chitosan-Ag (CS-Ag) NCs developed by Ganainy *et al.* exhibited significant antiviral activity against Alfalfa Mosaic Virus (AMV) in pepper plants. When applied topically 24 hours after inoculation, AMV infection was decreased by 90–91% at 400 ppm for CS NPs and 200 ppm for CS-Ag NCs.^231^ Xie *et al.* realized two bimetallic AgCu NCs–Ag_2_Cu_2_O_3_ and AgCuO_2_ that have remarkable antiviral capabilities; achieving a 6-log reduction of the Q beta (Qβ) bacteriophage. Notably, the material's ability to efficiently prevent virus transmission both day and night is demonstrated by the 7.5 log inactivation, resulting from its enhanced antiviral activity under visible light.^232^ Demchenko *et al.* developed PLA-Ag-PEI NCs that strongly inhibit viruses such as herpes simplex virus type 1, influenza A virus, and adenovirus serotype 2.^233^

#### 3.4.1 Ag based nanocomposites *vs.* SARS CoV-2

During the covid pandemic, Agnol *et al.* realized thermoplastic polyurethanes and Ag NCs (TPU/AgNPs) that exhibited potent antiviral properties against SARS-CoV-2, achieving an inactivation yield exceeding 99.0%.^234^ Similarly, Assis *et al.* made SiO_2_–Ag NCs immobilized on a polymeric ethyl vinyl acetate matrix that demonstrated strong antiviral properties against SARS-CoV-2.^235^ Additionally, Morozova *et al.* reported the antiviral properties of silver-based nanomaterials against SARS-CoV-2. To target SARS-CoV-2 and lower the possibility of viral resistance, a hybrid strategy was proposed, utilizing Ag-2S for RNA degradation and Ag nanoparticles to interfere with antigen interactions.^236^

### 3.5 Drug delivery

Ag nanoclusters (NCs) have proven to be highly efficient carriers for drug delivery due to their large surface area, biocompatibility, and customizable properties. To achieve effective targeted delivery, these NCs can be tailored to precisely control drug release, target specific cells or tissues, and minimize unwanted side effects. They function through mechanisms such as controlled release, enhanced cellular uptake, and selective targeting of specific cells.^237,238^

Recently, Hanna *et al.* synthesized a pH sensitive silver nanocomposite (SNCs) that worked as efficient biodegradable carriers for controlled intestinal delivery of 5-fluorouracil.^239^ Romdoni *et al.* developed Fe_3_O_4_@SiO_2_-Ag NPs, which demonstrated strong potential as a drug delivery system attributed to their super paramagnetic nature and ability to load anticancer drugs like epirubicin (EPI).^240^ In a recent study, Bertão, *et al.* created a zeolite-based delivery system, Ag_4_(5-FU)@Y, that offered a dual-function drug delivery platform combining antimicrobial silver (Ag^+^) and antineoplastic 5-fluorouracil (5-FU).^241^

Similarly, Mahanty *et al.* developed a biosurfactant (BS)-stabilized Ag NPs that offered enhanced drug delivery applications in combating antimicrobial resistance (AMR).^242^ Meligy *et al.* highlighted silver and gold NP-based chitosan nanocomposites that offered an efficient drug delivery platform for cancer treatment.^243^ Interestingly, Khafaga *et al.* synthesized a zinc oxide–superparamagnetic iron oxide–silver nanocomposite through green methods, which served as a nanocarrier to improve the anticancer efficacy of sorafenib.^244^

### 3.6 Biosensing

Ag NCs are being exploited as efficient biosensors for the detection of numerous biomolecules^245^ such as glucose,^246^ proteins,^247^ enzymes,^248^ cholesterol,^249^ and DNA.^250^ These materials enhance biosensing capabilities through mechanisms such as localized surface plasmon resonance (LSPR) and electrochemical signal amplification.

Recently, Mahmudin *et al.* described an easy method to detect *Escherichia coli* bacteria using Ag NPs-based localized surface Plasmon resonance (LSPR) biosensors.^251^ Pektaş *et al.* realized a novel amperometric glucose biosensor by modifying a carbon paste electrode (CPE) using green-synthesized WT-AgNPs derived from waste tea.^252^ Interestingly, Li *et al.* developed a flexible biosensing platform utilizing hollow Prussian blue NCs with ultra-small Ag NPs (Ag-HPB), possessing enhanced electrical conductivity and enzyme loading capacity. This platform demonstrated outstanding biosensing performance, with a sensitivity of 24.37 μA mM^−1^ cm^−2^ for glucose and a low limit of detection (LOD) of 2.28 pg mL^−1^ for trichlorfon (TCF).^253^ Saadh *et al.* incorporated Ag-Cu NPs into polyaniline nanotubes (Ag-Cu@PANI) that demonstrated efficient electrochemical detection of dopamine and hydroquinone simultaneously, with detection limits of 0.46 μM and 0.23 mM respectively.^254^

Similarly, Kim *et al.* reported casein hydrolysate peptides-functionalized Ag NPs (CHPs@AgNPs), which exhibited a colorimetric response to AGAs such as streptomycin, producing visible colour changes from yellow to orange, with absorbance peaks at 405 and 520 nm.^255^ Selimoglu *et al.* presented an interesting study where they created Ag NPs-doped graphene-based biosensor for procalcitonin, having LOD as low as 0.55 ng mL^−1^.^256^ Sukjee *et al.* detected the EV71 virus using an Ag NPs-based biosensor with a detection limit of 0.0001 PFU mL^−1^ in PBS and 0.001 PFU mL^−1^ in serum.^257^

### 3.7 Wound healing

Due to the numerous medicinal benefits of silver nanocomposites, there has recently been a notable increase in their applications for wound healing.^258,259^ These nanocomposites release silver ions that fight against the bacteria, fungi and viruses to prevent infections that can potentially slow down the wound recovery process. These silver-based nanocomposites ensure rapid wound healing by reducing inflammation, promoting cell growth and migration. As a result, silver nanocomposites can be integrated into wound dressings to create an optimal healing environment for treating cuts, burns, and wounds, while effectively preventing infection.^260–262^

For example, Aldakheel *et al.* opted microwave irradiation to fabricate Ag NPs, which were then loaded onto chitosan grafted PVA hydrogel to investigate their wound-healing ability in both *in vivo* and *in vitro* rat models. Additionally, they showed notable antibacterial activity against *S. aureus* and *E. coli*.^263^ Saghafi *et al.* reported the use of bromelain and Ag NPs incorporated into polycaprolactone/chitosan nanofibers (PCL/CS-Ag NPs-BRO) as a dressing for wound-healing applications. Interestingly, the addition of bromelian and Ag NPs notably improved the tensile strength and antibacterial activity of the nanofibers.^67^ In a similar study, Zhang *et al.* (2024) developed electrospun polyasparthydrazide nanofibers embedded with Ag nanoparticles (PAHy/Ag NPs) for wound healing applications. The resulting nanofiber hydrogel mat demonstrated an improved silver release rate of 9.4 ± 1.1% and showed strong antibacterial activity, killing 99.99% of both *E. coli* and *S. aureus*. This makes it a promising candidate for use in dressing materials for treating infected wounds, promoting collagen deposition at the wound site.^264^ Recently, Lakkim *et al.* addressed the wound healing potential of green-synthesized Ag NPs in mince using the excision wound model in Balb/C mice. Herein, the group observed increased collagen, DNA and protein content in wound samples, making it an efficient antioxidant compound for cutaneous wound treatment as a medicine or ointment.^265^ In 2024, Gawad *et al.* developed an efficient antimicrobial wound-healing substance using Ag NPs embedded natural hydrogel for rats with more than 98% wound area contraction in only 2 weeks.^266^

Recently, Vijayakumar *et al.* prepared Ag NP conjugated probiotic bacteria and investigated its bacterial growth inhibition and wound-treating capability. They observed strong antibacterial activity against pathogens and excellent wound closure of 96% through an *in vitro* scratch-wound assay.^267^ A novel fibrin/chitosan incorporated Ag nanocomposite was fabricated by Sanmugam *et al.* to level up the antibacterial and wound healing activity. The prepared nanocomposite exhibited antibacterial activity against *P. aeruginosa*, *E. coli*, *S. aureus* and *L. bulgarius* pathogens.^268^ Muneeswaran *et al.* prepared chitosan/Ag (CS/Ag) nanocomposites and investigated their microbial inhibition activity against *P. aeruginosa*, *E. coli*, *K. pneumoniae* and *S. typhi*. They observed that CS/Ag nanocomposite outperformed Ag NPs by exhibiting higher biofilm inhibition against *P. aeruginosa* and *K. pneumoniae* while also demonstrating cyto-compatibility with L929 mouse fibroblast cells. Notably, it enhanced cell migration by wound gap closure, making it a suitable wound healing agent for drug-resistant bacterial wound infections.^200^

Recently, Kodasi *et al.* prepared Chitosan-Ag nanocomposite and investigated its catalytic, anticancer, wound healing and antioxidant properties. The group tested normal (L929), lung cancer (A549) and oral cancer (KB-3-1) cell lines to access the anticancer activity, with promising IC_50_ values of 83.52 μg mL^−1^, 66.74 μg mL^−1^ and 75.11 μg mL^−1^ respectively.^269^ Exploiting the biocompatibility and antimicrobial property of silver, Amiri *et al.* developed an Ag NP-based hydrogel nanocomposite for efficient wound healing process. Herein, they accessed the wound healing capacity through the rat splinted wound method and evaluated wound infection prevention through the rat subcutaneous infection model.^270^ In a similar manner, Arghand *et al.* used eugenol coated Ag NPs embedded in an alginate–chitosan nanocomposite which demonstrated enhanced wound healing compared to the alginate–chitosan nanocomposite alone.^271^

Interestingly, Nguyen *et al.* incorporated biosynthesized Ag NPs into passion fruit peel pectin/chitosan biofilm to elevate their antibacterial and wound healing properties, with 100% wound closure after 15 days.^272^ Farazin *et al.* fabricated a flexible self-healing nanocomposite that offered rapid wound healing capabilities. Herein, to fabricate the system, they opted gelatin, acrylic acid and tannic acid as a matrix with ZnO and hollow Ag NPs.^273^ Recently, silver and gold nanoparticles were designed and developed using *A. macleodii* secreted exopolysaccharide (EPS) that exhibited excellent cell migration, contributing to a rapid wound healing.^274^ In 2023, Ebrahimzadeh *et al.* employed quercetin extract for the green synthesis of Ag NPs and studied the *in vivo* and *in vitro* antileishmanial activity. They displayed a promising *in vitro* IC_50_ value of 125 μg mL^−1^ against promastigotes. *In vivo*, the infected BALB/c mice were treated with topical application for 21 days, making them a promising antileishmanial drug.^275^

## 4. Environmental applications

Over many years, modernization and industrialization have significantly contributed to the alarming levels of pollutants in the air, water and soil. Additionally, they are also opted for the treatment of various water pollutants such as heavy metals, dyes, and organic contaminants. Furthermore, they are widely utilized in filtration systems to target airborne microbes and pollutants, helping to provide cleaner air.^226,276^ Moreover, these nanocomposites could also be used to eliminate organic pollutants or neutralize heavy metals contaminating the soil. Overall, silver nanocomposites hold great potential in improving the environmental conditions by removing, reducing or utilizing harmful substances offering better water, air and soil quality.^10,277,278^

Elevated levels of particulate matter, coming from vehicles, construction sites, burning of fossil and industrial emissions can severely threat human health. Many silver-based nanocomposites have been proved to be effective air filters for this purpose.^279^ For example, La *et al.* designed an excellent antibacterial particulate matter filter by integrating Ag/graphene nanocomposite onto textile material. The synthesized material offered a remarkable particulate matter removal of 98.5% along with high antibacterial activity against *E. coli* bacteria.^280^ Similarly, Yontar *et al.* developed special filter papers coated with green synthesized Ag and PVA nanocomposite that successfully enhanced the mechanical and antibacterial attributes of the filters.^281^

Not only as air filters, many Ag NCs were also reported as great photocatalyst for the degradation of many volatile organic compounds (VOCs) such as alcohols,^282^ aromatic^283^ and aliphatic hydrocarbons,^284^ aldehydes,^285^ ketones^286^*etc.* For example, Wanwong *et al.* reported a promising multifunctional air filter consisting of electrospun silk nanofiber loaded with Ag-doped TiO_2_. The reported system efficiently filtered about 99% of particulate matter, exhibited high antibacterial properties and demonstrated high photodegradation of formaldehyde.^287^ Similarly, Sboui *et al.* opted a simple method to deposit photocatalytic Ag–AgCl/TiO_2_ over cellulose film that successfully degraded a variety of VOCs such as ethanol, 1-propanol, 1-butanol, propylamine and propanethiol in gas phase under sunlight.^288^

Additionally, many researchers have reported the use of silver nanocomposites for CO_2_ capture and conversion.^64,289^ For example, by employing MOFs in their work, Liu *et al.* produced two heterogenous silver-based nanocomposites; core–shell and corner, which exhibited outstanding photocatalytic activity for CO_2_ adsorption and reduction reaction under irradiation. Herein, the corner MOF-Ag NC outperformed the core–shell MOF-Ag NC, attributed to its higher surface area to volume ratio.^290^ In a similar study, Nosrati *et al.* developed ternary and quaternary hybrid photocatalytic systems using graphene oxide, TiO_2_, Ag_2_O and arginine (GO–TiO_2_–Ag_2_O and GO–TiO_2_–Ag_2_O–Arg). Herein, the developed photonanocatalyst efficiently captured CO_2_ and reduced it to methanol under UV and visible light.^291^ Interestingly, Li *et al.* highlighted the application of ZnO/Ag/g-C_3_N_4_ nanocomposites for excellent photoactivated gas-sensing activity for NO_2_ detection.^292^

Apart from their application in dye degradation, many silver nanocomposites are very versatile in sensing and decomposing of various pesticides that pollute the water sources. For example, Singh *et al.* developed highly efficient Ag nanocomposites that exhibited a removal efficiency ranging from 64% to 88.5% for commonly used organophosphate pesticides like chlorpyrifos, malathion, dichlorvos and profenofos.^293^ Similarly, Chinnappa *et al.* green synthesized rGO–AgNP NCs and investigated the photocatalytic system for organophosphate chlorpyrifos pesticide degradation.^294^ Another recent study by Zheng *et al.* described the photocatalytic destruction of nitenpyram pesticide using Ag@AgCl/ZnAl-LDH nanocomposites achieved only in 45 min.^295^ Veerakumar *et al.* designed and developed palladium, silver NPs embedded on ZnO nanostars *via* microwave-hydrothermal method and studied their photocatalytic degradation activity for carcinogenic pesticides such as methyl parathion and herbicides like pendimethalin and trifluralin.^296^

Moreover, many studies also revealed the excellent detection capabilities of pesticides through Ag-based nanocomposites.^297–299^ Meanwhile, many recent researches targeted the heavy metal ion detection application in water and soil.^300^ For example, silver/graphene oxide nanocomposite developed by Dat *et al.* offered high Hg^2+^ sensitivity in water, attaining a limit of detection as low as 19.06 ± 0.42 μg L^−1^. Additionally, the developed nanocomposite attained 100% crystal violet dye removal through adsorption mechanism.^301^ Similarly, Şahin *et al.* removed heavy metal ions such as Ni^2+^, Cu^2+^, Pb^2+^, Cd^2+^ from natural water samples by employing Ag NPs and magnetic nanoparticles/nanocomposites.^302^

Interestingly, Shehawy *et al.* realized eco-friendly Ag NPS and studied their adsorbent property for removal of heavy metal such as Fe, Mn, Zn and Cu, achieving an excellent removal efficiency of 97.1% for Fe.^303^ Similarly, Amini *et al.* was able to extract trace amounts of heavy metals such as Ni^2+^, Cu^2+^, Mn^2+^, Cr^2+^ and Cd^2+^ from water and rice samples.^304^ Recently, Boas *et al.* developed peptide stabilised Au and Ag NPs that exhibited change in absorbance through a pH-dependent system for the selective and sensitive detection of Hg^2+^, Fe^2+^ and Mn^2+^.^305^ Moreover, many Ag NCs have been efficiently used as sensors for detecting heavy metal ions, nitrogen containing inorganic species, phenolic compounds, pharmaceuticals, nitroaromatics, natural and synthetic estrogens and more.^306^

## 5. Industrial applications

### 5.1 Agriculture

Agriculture is a vital sector for a nation's survival, as it ensures food supply for the population. Over time, the rising demand for food and related products has surged the focus on improving crop production and reducing agricultural losses. Ag NPs have been widely used to enhance seed germination, promote plant growth, and improve various crop development factors. For instance, Hojjat *et al.* investigated the effect of Ag NPs on lentil seed germination. Upon exposure to Ag nanoparticle, a significant enhancement in seedling growth, seed germination and mean germination time was observed in lentil seeds. Also, they observed that the exposed lentil seeds exhibited higher drought tolerance.^307^ Similarly, Antunes *et al.* explored how hyaluronic acid-stabilized Ag NPs (HA-AgNPs) worked as a seed priming agent to impact seed germination in lettuce (*Lactuca sativa* L.).^308^ Also, Rahman *et al.* experimented the use of Ag NPs for seed germination and growth performance of pea (*Pisum sativum*).^309^ For example, Ansari *et al.* fabricated Ag NPs by using Neem leaf extract (*Azadirachta indica*) and investigated their effect on tomato plant.^310^ In their recent study, Sambangi *et al.* employed biogenically synthesized Ag NPs to investigated chickpea plant growth and development. Streptomyces-mediated Ag NPs significantly improved the quality of chickpeas by promoting plant growth traits, nitrogen fixation, boosting defense enzyme activity, increasing yield as well as enhancing Fe, Zn, Mn and K contents.^311^

Khan *et al.* employed pistachio seed coat waste to synthesize Ag NPs, which were then sprayed on eggplant and investigated for their effect.^312^ Interestingly, many recent researches have highlighted the use of Ag-based NCs as a quick and easy electrochemical sensor for detection of macronutrients such as nitrogen, potassium, and phosphorus in soil or water to check and improve the agricultural conditions of soil and water in real-time.^313^

### 5.2 Food packaging and quality check

Over decades in the food industry, the research on food packaging hold pivotal importance to ensure safe good quality food. Researchers are constantly working to design materials that hold good mechanical strength and stability while having biodegradability and antibacterial properties, in order to extend the shelf life of the packaged food. Toxicity concerns due to plastic packing have paved way for researchers to explore natural biopolymers-based metal nanocomposites as packaging materials.^314–319^

For example, Guerraf *et al.* employed cellulose fibers, conducting polymers and Ag NPs to fabricate a nanocomposite, reliable to be used as an active food packaging material.^320^ Similarly, Liu *et al.* designed and developed soluble soyabean polysaccharide-based Ag NPs incorporated nanocomposite, that possessed improved UV-barrier and thermal properties.^321^ Recently, Abdallah *et al.* used agricultural biowaste to synthesize Ag NPs, which when entrapped into polyurethane nanofibers, notably enhanced their antibacterial and antioxidant attributes, paving way for more improved food safety and storage.^322^ Recently, Yang *et al.* realized the novel green synthesis of P. cocos polysaccharide as a stabilizing agent for Ag NPs that demonstrated strong antibacterial activity. Subsequently, they incorporated these nanoparticles into chitosan that prolonged the shelf life of strawberries while maintaining their quality.^323^

In addition, Yaqoob *et al.* designed and developed an Ag NPs incorporated bio-composite that demonstrated excellent antioxidant and antimicrobial properties.^324^ Novel bacteriocin assisted Ag NPs were realized by Sharma *et al.* and then coated onto cellulose paper to study their use as a packaging material.^325^ In a similar manner, Biswal *et al.* biosynthesized Ag NPs that displayed remarkable antibacterial and antioxidant properties that can be exploited for food packaging applications.^326^ Majumder *et al.* prepared a protein-based silver nanocomposite film that improved the water barrier characteristics of soy protein isolate making them suitable for storing high-moisture food products.^327^

Similarly, Amrutha *et al.* opted a one-pot method to synthesized Ag NPs using PVA/MC cross-linked and uncross-linked blends and was analysed for their thermal, mechanical and biomedical properties for food packaging application.^328^ In a similar manner, Li *et al.* developed antibacterial microcapsules consisting of Ginkgo biloba essential oil as the core with chitosan and gelatin as the capsule material, which were later on modified using green synthesized Ag NPs.^329^ In an intriguing study, Ragab *et al.* enhanced the optical, thermal, mechanical, electrical, and antibacterial properties of PVA–chitosan by incorporating biosynthesized Ag NPs. This resulted in the creation of a nanocomposite with potential applications in food storage.^330^ Recently, Pandian *et al.* developed a green synthesized Ag NPs and decorated them to fabricate a nanocomposite film, Ag NP/MCC/starch/whey protein, that can be employed as an antibacterial food packaging film in order to extend the shelf life of perishable foods by fighting food pathogens.^331^

In food industry, apart from food packaging, the freshness of food is also essential. Despite being stored efficiently, many environmental factors, namely pH, moisture, presence of oxygen, temperature *etc.* can potentially affect the food products and degrade their nutritive values.^332^ For example, Li *et al.* decorated pectin/gelatin films with curcumin and Ag NPs to prepare a renewable biomass-based food packaging material that possessed great antibacterial activity, mechanical strength, antioxidant activity and hydrophilicity.^333^

Similarly, Xu *et al.* designed a wearable glove sensor for visual identification of tetracycline antibiotics, whose excessive use can cause significant health issues. They combined Ag nanoclusters with an europium-based material to create a fast and highly responsive tetracycline sensor with a notable fluorescent color change. The synthesized nanosensor demonstrated a low detection limit of 10.5 nM, along with high sensitivity and quick response times.^334^ In 2024, Wang *et al.* reported a core–shell Au@Ag nanoparticle system sensitive to the presence of acrylamide, a human carcinogen that can potentially damage the human nervous and reproductive system.^335^ Similar to this, Anh *et al.* developed a highly sensitive electrochemical Ag-core@Fe_3_O_4_ nanosensor for Furazolidone detection in real food samples, excess of which can cause serious antibiotic residues and environmental pollution.^336^

In a recent study, Wang *et al.* designed 4-aminobenzenethiol-functionalized Au@Ag core–shell nanosensor (Au@Ag-4ABT NP) for detecting the presence of carbendazim (CBZ) in food samples.^337^ On similar notes, Wang *et al.* designed a novel 2D Au@Ag nanodot array and investigated fruit juices for pesticide contamination.^338^ In a similar way, Parnsubsakul *et al.* developed an eco-friendly and disposable AgNP-BNC paper composites that detected pesticides namely, aminothiophenol and methomyl on fruit surface.^339^

### 5.3 Textiles

Leveraging the antimicrobial properties of silver, many researchers are working to enhance textiles with these medicinal benefits. These advanced fabrics can be used not only in the healthcare sector for creating antibacterial surgical gowns, scrub suits, and protective clothing, but also in everyday items such as bedding, tablecloths, curtains, and more.^340^ For example, Mondal *et al.* designed antimicrobial fabric by incorporating Ag NPs-chitosan nanocomposite coating on cotton fabric.^341^ Similarly, Montemurro *et al.* achieved a long-lasting antibacterial fabric through novel polyoxometalate-modified silver nanocomposites suggesting its wide utility in multiple sectors.^342^ Phyto-chemical synthesized Ag NPs were coated on cotton and wool fabrics by Lite *et al.* and investigated their antimicrobial properties against bacteria and fungi.^343^ Interestingly, Wu *et al.* fabricated a durable antibacterial fabric pertaining anti-UV characteristics *via* grafting lipoic acid-modified amino compound and depositing Ag NPs over cellulose.^344^ In 2023, Plé *et al.* functionalized cotton textile with photoinduced Ag@polymer coating to incorporate antimicrobial activity into fabrics to address microbial proliferation.^345^ Green synthesized Ag NPs using mullein extract were deposited on nylon fabric by Kiakhani *et al.* to produce coloured fabrics possessing antibacterial and dyeing properties.^346^

Additionally, many silver-based nanocomposites have been employed for developing smart textiles.^347–349^ One such research was performed by Naysmith *et al.* where they coated polypyrrole-conjugated green synthesized Ag NPs that resulted in a low electrical resistance (9.56 × 10^1^ Ω per sq.) conductive textile fabric.^350^ Similarly, İlhan *et al.* investigated the electromagnetic interference shielding and antibacterial activity of Ag nanocomposite treated yarns.^351^ In 2023, Jagadeshvaran *et al.* highlighted the use of Ag NPs deposited cotton core with CNT shell for blocking electromagnetic radiations *via* an absorption–reflection–absorption approach.^352^

Moreover, some functionalized textiles have been employed for environmental remediation as well. For example, Gao *et al.* developed polypyrrole-silver/silver chloride contained multifunctional fabric that was used for photocatalytic destruction of organic pollutants (Rh B, MB, MO) and exhibited antibacterial activity.^353^ In 2023, La *et al.* fabricated an antibacterial cloth filter using Ag/graphene-integrated non-woven polypropylene textile. The synthesized material exhibited high particulate matter removal efficiency of 98.5% as well as high antibacterial activity against *E. coli*, making it an efficient air-pollutant filtering system.^280^

## 6. Conclusion

In summary, silver-based nanocomposites have proven to be highly versatile and impactful across various fields, driving significant advancements in material science. Their multifunctional properties—such as stability, reusability, and biocompatibility—along with exceptional catalytic, antimicrobial, and environmental remediation capabilities, make them invaluable tools for tackling contemporary challenges. Whether used in catalytic organic processes, biomedical applications, or environmental cleanup, these nanocomposites consistently surpass conventional materials in both efficiency and specificity. Furthermore, their applications in agriculture, food packaging, and textiles have spurred new innovations, enhancing plant health, food preservation, safety, and material functionality. As research progresses, silver-based nanocomposites are expected to continue playing a key role in technological advancements, providing promising solutions in fields such as environmental protection and healthcare. The developments from 2019 to 2024 presented here emphasize the increasing versatility of these materials, reinforcing their significance in the future of nanotechnology-driven progress.

## Data availability

The data supporting the findings of this study are available upon reasonable request from the corresponding author.

## Conflicts of interest

All the authors declared that they have no conflict of interest.
